# Hypoxia-induced P4HA1 overexpression promotes post-ischemic angiogenesis by enhancing endothelial glycolysis through downregulating FBP1

**DOI:** 10.1186/s12967-024-04872-x

**Published:** 2024-01-18

**Authors:** Yating Xu, Di Xia, Kai Huang, Minglu Liang

**Affiliations:** 1 Clinic Center of Human Gene Research, Union Hospital, Tongji Medical College, Huazhong University of Science and Technology, 1277 Jiefang Ave, Wuhan, 430022 China; 2 Hubei Key Laboratory of Metabolic Abnormalities and Vascular Aging, Huazhong University of Science and Technology, Wuhan, China; 3 Hubei Clinical Research Center for Metabolic and Cardiovascular Disease, Huazhong University of Science and Technology, Wuhan, China; 4 Department of Cardiology, Union Hospital, Tongji Medical College, Huazhong University of Science and Technology, Wuhan, China

**Keywords:** P4HA1, α-Ketoglutarate, TET2-FBP1 pathway, Glycolysis, Angiogenesis

## Abstract

**Background:**

Angiogenesis is essential for tissue repair in ischemic diseases, relying on glycolysis as its primary energy source. Prolyl 4-hydroxylase subunit alpha 1 (P4HA1), the catalytic subunit of collagen prolyl 4-hydroxylase, is a glycolysis-related gene in cancers. However, its role in glycolysis-induced angiogenesis remains unclear.

**Methods:**

P4HA1 expression was modulated using adenoviruses. Endothelial angiogenesis was evaluated through 5-ethynyl-2′-deoxyuridine incorporation, transwell migration, and tube formation assays in vitro. In vivo experiments measured blood flow and capillary density in the hindlimb ischemia (HLI) model. Glycolytic stress assays, glucose uptake, lactate production, and quantitative reverse transcription-polymerase chain reaction (RT-PCR) were employed to assess glycolytic capacity. Transcriptome sequencing, validated by western blotting and RT-PCR, was utilized to determine underlying mechanisms.

**Results:**

P4HA1 was upregulated in endothelial cells under hypoxia and in the HLI model. P4HA1 overexpression promoted angiogenesis in vitro and in vivo, while its knockdown had the opposite effect. P4HA1 overexpression reduced cellular α-ketoglutarate (α-KG) levels by consuming α-KG during collagen hydroxylation. Downregulation of α-KG reduced the protein level of a DNA dioxygenase, ten–eleven translocation 2 (TET2), and its recruitment to the fructose-1,6-biphosphatase (FBP1) promoter, resulting in decreased FBP1 expression. The decrease in FBP1 enhanced glycolytic metabolism, thereby promoting endothelial angiogenesis.

**Conclusions:**

Hypoxia-induced endothelial P4HA1 overexpression enhanced angiogenesis by promoting glycolytic metabolism reprogramming through the P4HA1/α-KG/TET2/FBP1 pathway. The study’s findings underscore the significance of P4HA1 in post-ischemic angiogenesis, suggesting its therapeutic potential for post-ischemic tissue repair.

**Supplementary Information:**

The online version contains supplementary material available at 10.1186/s12967-024-04872-x.

## Background

Angiogenesis, the process of forming new blood vessels from pre-existing ones, plays a crucial role in various biological processes such as wound healing, ischemia, and cancer metastasis. Timely angiogenesis is essential for protecting tissues from injury in ischemia-related diseases, such as peripheral arterial diseases, stroke, and myocardial infarction [1]. Therefore, a comprehensive understanding of the mechanisms underlying angiogenesis is essential for identifying potential therapeutic targets for these diseases.

Angiogenic growth factors, including fibroblast growth factors, vascular endothelial growth factors, and angiopoietin, play pivotal roles in regulating angiogenesis. While many studies currently focus on these growth factors and associated signaling pathways [2], treatments targeting them still face challenges in terms of efficacy and specificity. Several angiogenic growth factors have been identified to induce angiogenesis by modulating cellular metabolism to ensure an adequate energy supply [3]. Recent research has highlighted the close relationship between metabolism and endothelial cell (EC) function [4, 5]. Therefore, targeting endothelial metabolism holds promise for enhancing the effectiveness of pro-angiogenic therapy. Despite the higher ATP production capacity of oxidative metabolism compared to glycolysis, ECs predominantly rely on glycolysis, accounting for up to 85% of their total ATP. This preference is attributed to the rapid ability of glycolysis to produce ATP and shield ECs from oxidative stress. Additionally, glycolysis becomes more significant under hypoxic conditions, making ECs more resilient to oxygen deprivation [4, 5]. Moreover, glycolysis plays a critical role in promoting EC migration, extension, and proliferation [6–8]. Knockdown of the rate-limiting enzyme in glycolysis, 6-phosphofructo-2-kinase/fructose-2,6-bisphosphatase 3 (PFKFB3), has been shown to impair blood vessel sprouting [9]. Despite the increasing focus on glycolysis in angiogenesis, further investigation is needed to elucidate the underlying mechanisms of glycolysis-induced angiogenesis.

Collagen prolyl 4-hydroxylase (P4H), an α-ketoglutarate (α-KG)-dependent dioxygenase, facilitates proline hydroxylation in collagen, promoting collagen synthesis by utilizing α-KG as a substrate and releasing succinate as a product [10]. P4H consists of two identical catalytic subunits and two identical β subunits (i.e., P4HB). The major isoform among the catalytic subunits is P4H subunit alpha 1 (P4HA1). Han et al. reported that P4HA1 promotes angiogenesis in glioblastoma multiforme by driving the transition of stem-like cells into tumor ECs through the P4HA1/COL6A1/CD31 pathway [11]. P4HA1 also enhances glioma neovascularization by facilitating the transition of glioma stem cells into ECs and the formation of vascular basement membrane [12]. While existing research on the relationship between P4HA1 and angiogenesis mainly focuses on its role in the transformation of tumor stem cells into ECs, the role of P4HA1 in post-ischemic angiogenesis and the underlying mechanisms remains unclear. Bioinformatics analysis has identified P4HA1 as a glycolysis-related gene in various cancers [13, 14], suggesting its potential involvement in glycolysis-induced angiogenesis. Therefore, there is a need to elucidate the role of P4HA1 in glycolytic metabolism reprogramming-induced endothelial angiogenesis.

Fructose-1, 6-biphosphatase (FBP1) serves as a rate-limiting enzyme in gluconeogenesis, negatively regulating glycolysis [15]. In clear cell renal cell carcinoma, the DNA dioxygenase ten–eleven translocation 2 (TET2) activates FBP1 expression, thereby suppressing glycolytic capability [16]. TET2 is an α-KG- and Fe (II)-dependent DNA dioxygenase that catalyzes the conversion of 5-methylcytosine to 5-hydroxy-methyl cytosine during gene expression regulation [17]. Importantly, cellular α-KG influences TET2 protein levels, and supplementation with α-KG can enhance TET2 protein levels [18, 19]. Nevertheless, the role of the TET2-FBP1 pathway in glycolysis-induced angiogenesis and its interaction with P4HA1 remain unexplored. In this study, we demonstrate that P4HA1 promotes angiogenesis by boosting endothelial glycolysis. Furthermore, it reveals that P4HA1 induces glycolytic metabolism reprogramming through the α-KG/TET2/FBP1 pathway. This study is the first to establish a connection between P4HA1 and the TET2-FBP1 pathway via α-KG as an intermediary, introducing the novel concept of the P4HA1/α-KG/TET2/FBP1 pathway. These findings may open new avenues for therapeutic strategies targeting P4HA1 to enhance angiogenesis in ischemia-related diseases, potentially improving clinical outcomes in conditions such as peripheral arterial diseases, stroke, and myocardial infarction.

## Methods

### Cell culture and adenovirus infection

Human umbilical vein endothelial cells (HUVECs) were isolated and cultured in EC culture medium (ECM, 1001, ScienCell, USA) at 37 °C with 5% CO_2_. To induce hypoxic conditions, HUVECs were incubated in an incubator (3131, Thermo Scientific, USA) with 1% O_2_ and 5% CO_2_ for specified durations. Human microvascular pericyte cells (PCs, CP-H169, Procell, Wuhan, China) were cultured in a pericyte cell culture medium (CM-H169; Procell). HUVECs and human microvascular PCs were infected with the indicated adenoviruses to achieve overexpression or knockdown of specific genes.

### Murine model of hindlimb ischemia (HLI)

All animal experimental procedures followed the Guide for the Care and Use of Laboratory Animals and received approval from the Institutional Animal Care and Use Committee of Huazhong University of Science and Technology. To induce HLI, C57BL/6J male mice (6 weeks old) were intraperitoneally administered 50 mg/kg sodium pentobarbital. The femoral artery was exposed, ligated proximally and distally, and the middle portion was excised. Gastrocnemius muscle injection with 100 µL adenovirus containing 10^9^ plaque-forming units was performed on the day of surgery. Blood flow was assessed using a laser Doppler system (Perimed, Stockholm, Sweden) before surgery and at 1, 3, 7, and 14 days post-surgery. At 14 days after surgery, mice were euthanized, and the gastrocnemius muscle was harvested and divided into two parts. One part was fixed in 4% paraformaldehyde overnight, embedded in paraffin, and sectioned. The other part was separated into endothelial and non-endothelial components using CD31-conjugated Dynabeads (11061D, Thermo Scientific, USA) according to the manufacturer’s instructions.

### In vivo spheroid-based angiogenesis assay

Female CB17 SCID mice (Charles River, Wilmington, MA, USA) were used for the spheroid-based angiogenesis assay. Spheroids consisting of HUVECs and human microvascular PCs, pre-transfected with adenoviruses in a 1:1 ratio, were generated following established protocols [20]. Spheroids were formed by culturing cells in hanging drops of ECM containing 0.24% methylcellulose (HY-125,861, MCE, China). Spheroids of HUVECs pre-transfected with adenoviruses Ad-NC, Ad-P4HA1, a combination of Ad-P4HA1 and Ad-FBP1, or a combination of Ad-P4HA1 and Ad-TET2 were generated using the same procedure. The spheroids were then harvested, washed, and centrifuged for collection. A plug mixture, consisting of Matrigel matrix (354234, BD Biosciences, USA), spheroids, fibrinogen (2 mg/mL, 341576, Sigma, USA), methylcellulose, EC basal medium, and thrombin (10602400001, Sigma, USA) was injected into the groin of the mice (500 µL total volume) following a previously reported method [21]. After 21 days, mice were euthanized, and the plugs were harvested, embedded in paraffin, and sectioned for immunofluorescent staining of CD31.

### Western blotting

Cellular proteins were extracted using RIPA lysis buffer (G2002, Servicebio, Wuhan, China), and protein concentrations were determined using a BCA kit (G2026, Servicebio, Wuhan, China). Subsequently, proteins were separated through electrophoresis and transferred onto polyvinylidene difluoride (PVDF) membranes (IPVH00010, Millipore, Burlington, Massachusetts, USA). After blocking with 5% non-fat milk, the membranes were incubated with specific primary antibodies at 4 °C overnight, followed by incubation with secondary antibodies for 1 h at 25 °C. Chemiluminescence reagent (G2014, Servicebio, Wuhan, China) was employed for signal detection using the Clinx imaging system (Shanghai, China). Primary antibodies against P4HA1 (1:1000, 12658-1-AP) and β-actin (ACTB, 1:1000, 20536-1-AP) were purchased from Proteintech (Wuhan, China), and against TET2 (1:1000, A5682), FBP1 (1:1000, A11664), P4HA2 (1:1000, A22150), P4HA3 (1:1000, A13767), P4HB (1:1000, A19239) from AbClonal (Wuhan, China). The primary antibody for hypoxia-inducible factor-1α (HIF-1α, GTX127309) was purchased from Gene Tex (Irvine, CA, USA). The primary antibody for Flag (1:1000, MA1-91878) was purchased from Thermo Scientific (USA). Gastrocnemius muscle tissue proteins were extracted using the same procedure followed by homogenization.

### Immunofluorescence staining

For antigen retrieval, paraffin-embedded gastrocnemius muscle sections were heated at 121 °C for 10 min. Sections were blocked with 5% donkey serum (G1217, Servicebio, Wuhan, China) and incubated with primary antibodies at 4 °C overnight. Finally, sections were incubated with fluorescent secondary antibodies for 1 h and DAPI (GDP1024, Servicebio, Wuhan, China) for 15 min. Immunofluorescence was visualized and imaged using a fluorescence microscope (Olympus, Tokyo, Japan). Antibodies used in the immunofluorescence assay were against P4HA1 (1:100, 12658-1-AP, Proteintech), NG2 (1:200, ab275024, Abcam, Cambridge, UK), Myosin (1:200, ab37484; Abcam, Cambridge, UK), CD31 (1:400, ab182981; Abcam, Cambridge, UK), and Flag (1:200, MA1-91878, Thermo Scientific, USA).

### Recombinant adenovirus construction

The gateway system was employed for adenovirus construction, where restriction enzymes and ligases were utilized to construct plasmids. The cDNAs of the genes of interest were cloned into the overexpression vector pENTRY-ccdB-T2-pcdh using the CMV promoter, while the shRNAs of the genes of interest were cloned into the knockdown vector pENTRY-kd-ccdB2 with the U6 promoter. The entry vector, pENTRY, and the destination vector, pDEST (Thermo Fisher Scientific, USA), underwent catalysis by Gateway LR Clonase II Enzyme (11791020, Thermo Fisher Scientific, USA) to generate adenoviral plasmids. These plasmids were then linearized with the PacI enzyme (FD2204, Thermo Fisher Scientific, USA) and transfected into HEK293A cells for recombinant adenovirus packing and amplification. Adenoviruses carrying P4HA1 (Ad-P4HA1) were constructed, with null viruses (Ad-NC) serving as controls for overexpression. An adenovirus shRNA against P4HA1 (Ad-shP4HA1) and an adenovirus carrying null shRNA (Ad-shNC) were constructed to achieve knockdown. The recombinant adenoviruses were purified using the CsCl gradient centrifugation method.

### 5-Ethynyl-2′-deoxyuridine (EdU) incorporation assay

HUVECs infected with the specified adenovirus were cultured with 0.1% EdU for 12 h. Subsequently, cells were stained using an EdU cell proliferation kit (C0075L, Beyotime, Shanghai, China) according to the manufacturer’s instructions, and nuclei were counterstained with DAPI.

### Transwell migration assay

HUVECs infected with the indicated adenovirus were seeded into the upper chamber of a transwell with a serum-free cell culture medium. The lower chamber was filled with ECM containing 5% fetal bovine serum. After 12 h, migrated cells were stained with crystal violet, and images were captured using light microscopy (Olympus, Tokyo, Japan).

### Tube formation assay

For the tube formation assay, 96-well plates were coated with Matrigel Matrix. Subsequently, 2 × 10^4^ HUVECs were seeded, and after 4 h, images of tube formation were captured using a light microscope.

### RNA sequencing (RNA-seq)

RNA-seq analysis was performed using the Illumina platform. Total RNA was extracted from HUVECs infected with Ad-P4HA1 or Ad-NC under normoxia and assessed for quality control. mRNA underwent enrichment, fragmentation, and cDNA library construction. The resulting cDNA library was utilized for RNA-seq on the Illumina PEI150 sequencing platform, involving bridge amplification and base calling. Raw RNA-seq data underwent filtering, adaptor removal, and mapping to the human genome. Bioinformatics analysis was conducted using the RStudio software, with R packages such as ‘DEseq2,’ ‘ggplot2,’ and ‘ggreple’ used for differential expression analysis and volcano plot creation. Genes meeting the criteria |log2 (fold change)| ≥ 1 and p-value ≤ 0.05 were considered differentially expressed. Gene Set Variation Analysis (GSVA) and Gene Set Enrichment Analysis (GSEA) of differentially expressed genes were performed and visualized using R packages ‘clusterProfiler,’ ‘GSVA,’ ‘limma,’ and ‘enrichplot.’

### RNA extraction and quantitative reverse transcription-polymerase chain reaction (RT-PCR)

Total RNA was extracted from cells using the TRIzol reagent (9108, Takara, Japan), and mRNA concentrations were determined with a Nanodrop (ND-LITE, Thermo Fisher Scientific, USA). Equal amounts of RNA were reverse transcribed to cDNA using the PrimeScript™ RT Reagent Kit (RR037A, Takara, Japan). RT-PCR was performed using SYBR Green Master Mix (Q121-02/03, Vazyme, Nanjing, China). The primers used were as follows:

- PFKFB3: F: 5′-TTGGCGTCCCCACAAAAGT, R: 5′-AGTTGTAGGAGCTGTACTGCTT.
- LDHA: F: 5′- ATGGCAACTCTAAAGGATCAGC-3′, R: 5′- CCAACCCCAACAACTGTAATCT-3′.
- GLUT1: F: 5′-GGCCAAGAGTGTGCTAAAGAA-3′, R: 5′-ACAGCGTTGATGCCAGACAG-3′.
- ALDOA: F: 5′-ATGCCCTACCAATATCCAGCA-3′, R: 5′-GCTCCCAGTGGACTCATCTG-3′.
- HK2: F: 5′-GAGCCACCACTCACCCTACT-3′, R: 5′-CCAGGCATTCGGCAATGTG-3′.
- P4HA1: F: 5′-AGTACAGCGACAAAAGATCCAG-3′, R: 5′-CTCCAACTCACTCCACTCAGTA-3′.
- FBP1: F: 5′-CGCGCACCTCTATGGCATT-3′, R: 5′-TTCTTCTGACACGAGAACACAC-3′.
- 18S: F: 5′-TTGACGGAAGGGCACCACCAG-3′, R: 5′-GCACCACCACCCACGGAATCG-3′.

### Measurement of glucose uptake, lactate production, cellular ATP, and cellular α-KG levels

Glucose assay kit (BC2505, Solarbio, Beijing, China), Lactate Assay Kit (A019-2, Nanjing Jiancheng Bioengineering Institute, Nanjing, China), α-Ketoglutaric Acid Content Assay kit (MAK054, Sigma-Aldrich, USA), and ATP Assay Kit (S0026, Beyotime, China) were used to measure glucose uptake, lactate production, cellular α-KG levels, and cellular ATP, respectively, following the manufacturer’s instructions.

### Glycolysis stress assay

A Seahorse XF96 Flux Analyzer (Seahorse Bioscience, Billerica, MA, USA) was utilized to assess glycolytic metabolism. HUVECs infected with adenovirus (2.5 × 10^4^ cells) were seeded in 24-well plates (V7-PS). The cells were incubated in a non-CO_2_ incubator for 1 h in an assay medium supplemented with 2 mM glutamine (HY-N0390, MCE, China). The extracellular acidification rate (ECAR) was measured upon glucose (20 mM, HY-B0389, MCE, China), oligomycin (2 µM, HY-N6782, MCE, China), and 2-deoxyglucose (2-DG, 50 mM, HY-13966, MCE, China) injections. Basal glycolysis and glycolytic capacity were calculated from the ECAR data.

### Chromatin immunoprecipitation assay (ChIP)

ChIP was performed using a ChIP assay kit (Millipore) following the manufacturer’s instructions. DNA fragments, produced by sonication, were incubated with antibodies against TET2 (MABE 462, Millipore, USA) and IgG (as a control) at 4 °C overnight. Enriched DNA fragments were detected by PCR with the following FBP1 ChIP primers: F:5′-GATCCCCGACCTTGTCTGAA-3′, R:5′-TCGCGGAAACCTTTAGACGC-3′.

### Dual-luciferase reporter assay

The indicated regions of the FBP1 promoter were obtained from genomic DNA by PCR. pGL3 Luciferase reporter vectors (Promega, Madison, WI, USA) were constructed with the FBP1 promoter region using restriction enzyme and ligase. HUVECs were infected with Ad-NC or Ad-P4HA1 and transfected with the indicated pGL3 plasmids and pRL-TK (P0372, Miaoling, Wuhan, China). After 48 h, a dual-luciferase reporter assay detection kit (RG027, Beyotime, Shanghai, China) was used to measure luciferase activity.

### Statistical analysis

All data are presented as means ± standard error of the mean. Statistical analysis was performed using GraphPad Prism (GraphPad Software, San Diego, USA). The Shapiro–Wilk test and Bartlett’s test were employed to confirm the normal distribution and homogeneity of variance in the data. Student’s t-test, one-way analysis of variance (ANOVA) with Bonferroni post-hoc test, and two-way ANOVA with Bonferroni post-hoc test were utilized to determine significant differences. Statistical significance was set at *p* < 0.05.

## Results

### P4HA1 is upregulated in HUVECs under hypoxia and in the HLI model

**Fig. 1:**
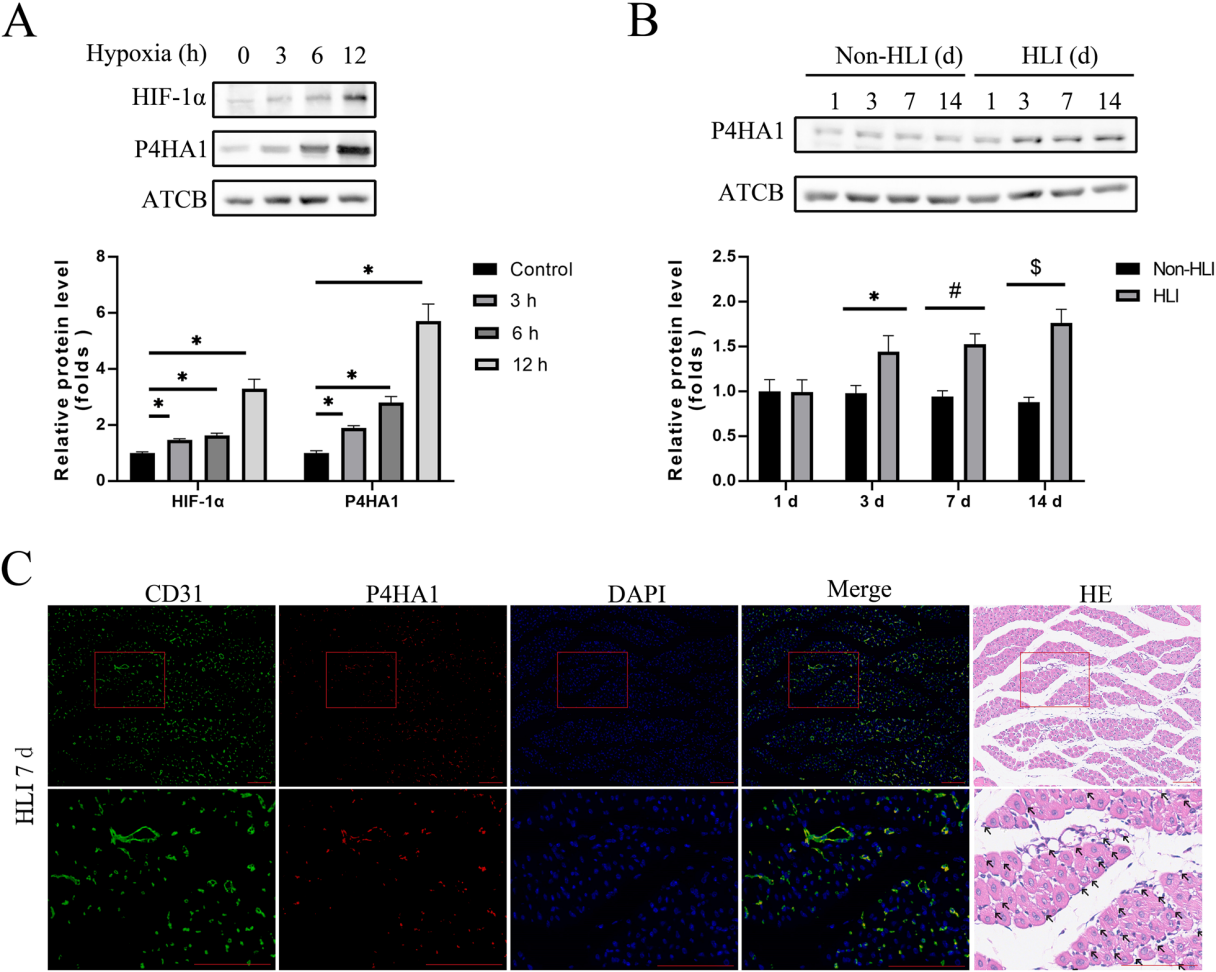
P4HA1 is upregulated in the HUVEC hypoxia model and ischemic muscle tissue. **A** Western blotting analysis of protein levels of HIF-1α, P4HA1, and ACTB in HUVECs under 0 h, 3 h, 6 h, and 12 h of hypoxia. Data were analyzed by one-way ANOVA followed by Bonferroni post hoc test (*n* = 3). *p < 0.05. **B** Western blotting analysis of protein levels of P4HA1 and ACTB in the gastrocnemius muscle of the HLI model and non-HLI mice. Data were analyzed by two-way ANOVA with Bonferroni post hoc test (*n* = 5). *p < 0.05 vs. the non-HLI group (3 d). ^#^*p* < 0.05 vs. the non-HLI group (7 d). ^$^*p* < 0.05 vs. the non-HLI group (14 d). **C** Hematoxylin-eosin staining and immunofluorescence staining of CD31 (green), P4HA1 (red), and DAPI (blue) in the gastrocnemius muscle tissue from the HLI model. Magnification: upper, 100×; lower, 400×. Scale bars: 100 μm. *HLI* hindlimb ischemia, *non-HLI* non-hindlimb ischemia, *HE* hematoxylin–eosin staining

P4H family proteins, namely P4HA1, P4HA2, P4HA3, and P4HB, were examined in HUVECs subjected to hypoxia. P4HA1 and HIF-1α exhibited upregulation during 3, 6, and 12 h hypoxia in a time-dependent manner (Fig. 1A). P4HA2 demonstrated slight upregulation after 12 h hypoxic exposure (Additional file 1: Fig. S1). However, the protein levels of P4HA3 and P4HB remained unchanged under hypoxic conditions (Additional file 1: Fig. S1). Western blot analysis of gastrocnemius muscle tissue lysates from the HLI model indicated an upregulation of P4HA1 at days 3, 7, and 14 (Fig. 1B). Immunofluorescence staining of gastrocnemius muscle tissues from the HLI model revealed the co-localization of P4HA1 with CD31, an EC marker (Fig. 1C). Vascular structures identified through hematoxylin-eosin staining aligned with CD31-positive regions in immunofluorescence, confirming its reliability (Fig. 1C). This observation lead to the conclusion that endothelial P4HA1 is upregulated under hypoxia and in the HLI model, suggesting its involvement in ischemia-induced angiogenesis.

### P4HA1 overexpression facilitates angiogenesis in vitro and in vivo

**Fig. 2:**
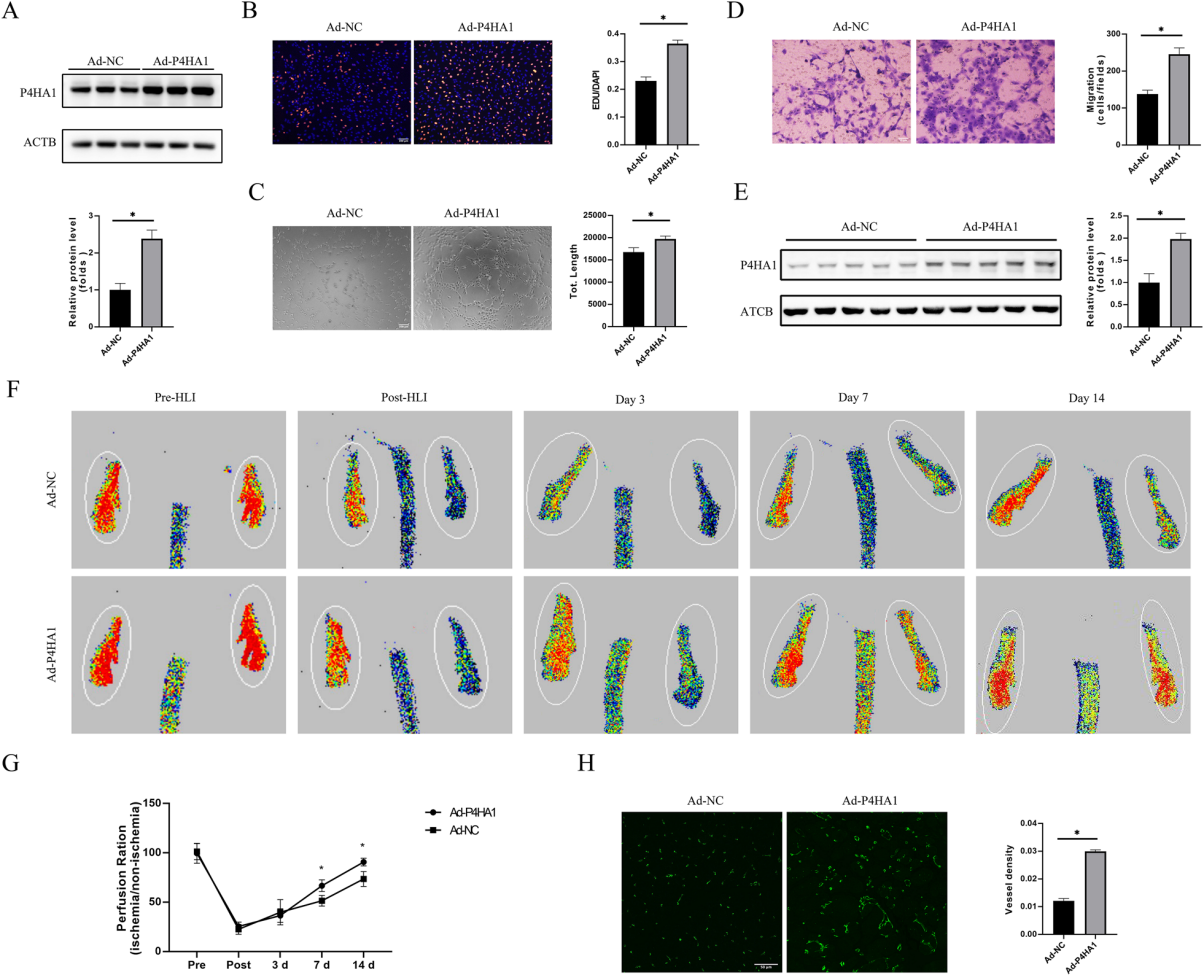
P4HA1 overexpression promotes angiogenesis in vitro and in vivo. **A** Validation of Ad-P4HA1 overexpression efficiency in HUVECs (*n* = 3). **B**–**D** After HUVECs were infected with Ad-NC and Ad-P4HA1 for 24 h, **B** EdU incorporation assay (magnification: ×100, scale bar: 100 μm), **C** tube formation assay (magnification: ×100, scale bar: 100 μm), and **D** transwell migration assay (magnification: ×400, scale bar: 50 μm) were performed (*n* = 3). **E** In vivo, P4HA1 overexpression was achieved by injecting purified adenoviruse into the gastrocnemius muscle on the first and seventh day after surgery. Overexpression efficiency of P4HA1 in gastrocnemius muscle of the HLI model (14 d) was detected and analyzed (*n* = 5). **F** Representative Doppler images of blood flow recovery after the HLI model with P4HA1 overexpression. **G** Statistical analyses of blood flow recovery in HLI model with P4HA1 overexpression (*n* = 5). **H** Immunofluorescence staining of CD31 (green) in the gastrocnemius muscle from the HLI model (14 d) with P4HA1 overexpression. magnification: ×400, scale bar: 50 μm. The fluorescence intensity was statically analyzed (*n* = 5). Data in **A**–**E**, **H** were analyzed using the Student’s t-test. Data in **G** were analyzed by two-way ANOVA with Bonferroni post hoc test. *p < 0.05. *NC* negative control, *HLI* hindlimb ischemia

To explore the effects of P4HA1 overexpression on the proliferation, migration, and tube formation of HUVECs, adenoviruses Ad-P4HA1 and Ad-NC were constructed. Western blotting confirmed that Ad-P4HA1 infection increased P4HA1 protein levels compared to the control group (Fig. 2A). The EdU incorporation assay indicated a significant increase in the number of EdU-positive cells, signifying enhanced cell proliferation due to P4HA1 overexpression under normoxia (Fig. 2B). The tube formation assay demonstrated that P4HA1 overexpression facilitated the formation of capillary-like structures and increased tube length under normoxic condition (Fig. 2C). Additionally, P4HA1 overexpression significantly augmented the number of migrating cells under normoxia (Fig. 2D).

An in vivo HLI model was established to investigate the effects of P4HA1 overexpression. Ad-P4HA1 and Ad-NC were injected into the gastrocnemius muscle of mice in the HLI model through local intramuscular injection on the first and seventh days post-surgery for in vivo P4HA1 overexpression. Western blotting confirmed the specific targeting of Flag-tagged P4HA1 to muscle tissue (Additional file 2: Fig. S2A). CD31-conjugated Dynabeads were used to isolate ECs from the gastrocnemius muscle, while the remaining suspension was designated as non-ECs. Western blotting confirmed the upregulation of P4HA1 in the gastrocnemius muscle and ECs following adenovirus Ad-P4HA1 infection (Fig. 2E, Additional file 2: Fig. S2B). The Flag-tag associated with adenovirus Ad-P4HA1 was predominantly expressed in the ECs of the gastrocnemius muscle (Additional file 2: Fig. S2C). Immunofluorescence revealed that Flag-tagged P4HA1 was mainly overexpressed in ECs and NG2-positive PCs rather than in myosin-positive skeletal muscle cells (Additional file 2: Fig. S2D). The data demonstrated that P4HA1 upregulation significantly increased perfusion at 7 and 14 days post-surgery compared to the control group (Fig. 2F, G). Immunofluorescence staining of gastrocnemius muscle tissues indicated that P4HA1 upregulation increased vessel density in the HLI model (Fig. 2H). In conclusion, P4HA1 upregulation promotes angiogenesis both in vitro and in vivo.

### P4HA1 knockdown inhibits angiogenesis in vitro and in vivo

**Fig. 3:**
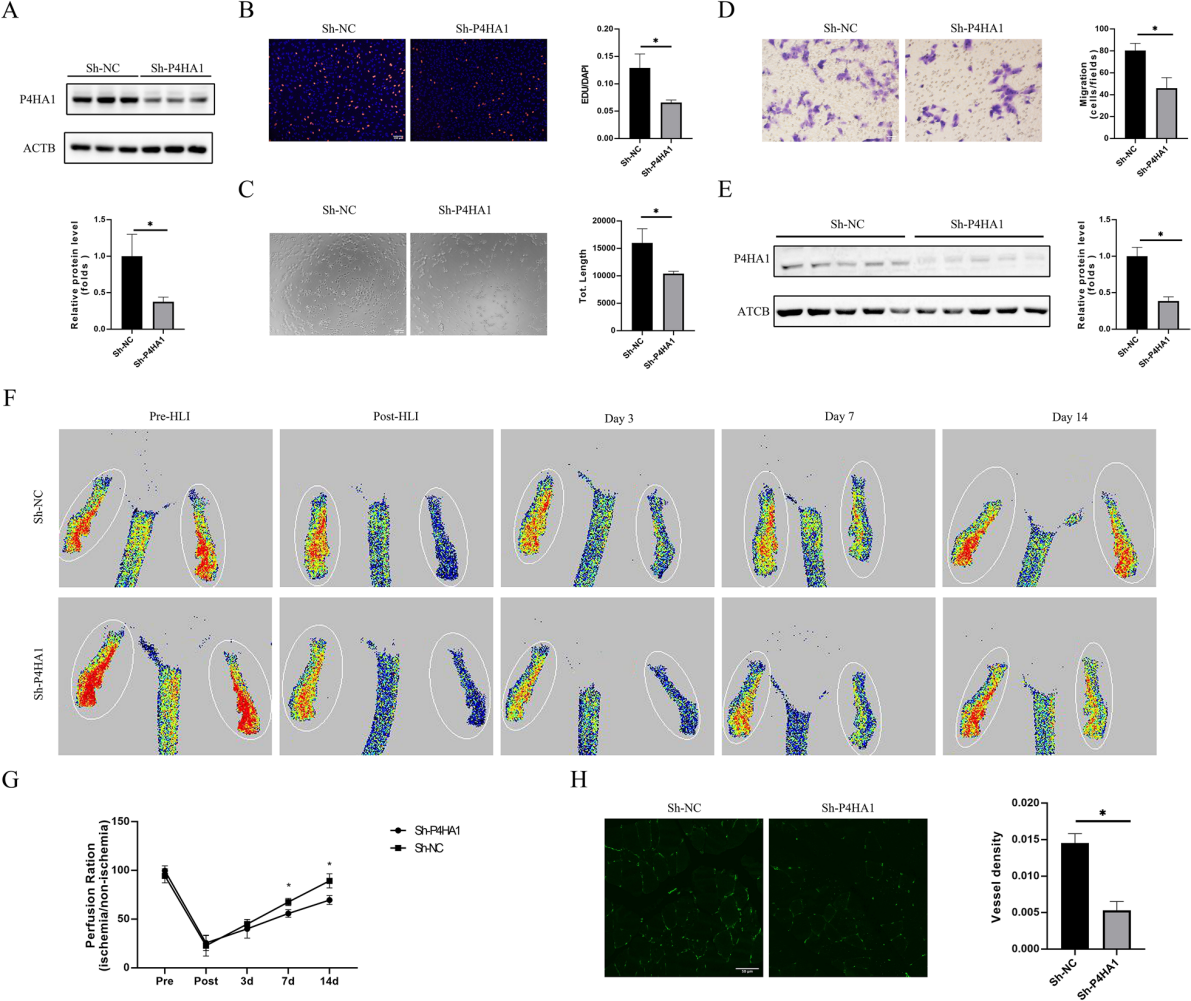
P4HA1 knockdown suppresses angiogenesis in vitro and in vivo. **A** Validation of knockdown efficiency of Ad-shP4HA1 in HUVECs (*n* = 3). **B**–**D** After HUVECs were infected with Ad-shNC and Ad-shP4HA1 for 24 h, **B** EdU incorporation assay (magnification: ×100, scale bar: 100 μm), **C** tube formation assay (magnification: ×100, scale bar: 100 μm), and **D** transwell migration assay (magnification: ×400, scale bar: 50 μm) were performed (*n* = 3). **E** In vivo, P4HA1 knockdown was achieved by locally injecting purified adenoviruse into the gastrocnemius muscle on the first and seventh day after surgery. The knockdown efficiency of P4HA1 in gastrocnemius muscle of the HLI model (14 d) was detected and analyzed (*n* = 5). **F** Representative Doppler images of blood flow recovery after HLI with P4HA1 knockdown. **G** Statistical analyses of blood flow recovery in the HLI model with P4HA1 knockdown (*n* = 5). **H** Immunofluorescence staining of CD31 (green) in the gastrocnemius muscle from HLI model (14 d) with P4HA1 knockdown. Magnification: ×400, scale bar: 50 μm. The fluorescence intensity was statistically analyzed (*n* = 5). Data in **A**–**E**, **H** were analyzed using the Student’s t-test. Data in **G** were analyzed by two-way ANOVA with Bonferroni post hoc test. *p < 0.05. *NC* negative control, *HLI* hindlimb ischemia

For in vitro and in vivo P4HA1 knockdown, we constructed Ad-shP4HA1, with Ad-shNC as the control. Western blotting confirmed that Ad-shP4HA1 infection effectively downregulated P4HA1 in HUVECs (Fig. 3A). The EdU incorporation assay demonstrated that P4HA1 knockdown significantly inhibited endothelial proliferation under normoxia (Fig. 3B). Similarly, P4HA1 knockdown impeded the tube formation capability of ECs under normoxia (Fig. 3C). The transwell migration assay indicated a suppressed migration capability of HUVECs following P4HA1 knockdown under normoxia (Fig. 3D). For the in vivo assay, we injected purified Ad-shP4HA1 and Ad-shNC into the gastrocnemius muscle of the HLI model on the first and seventh days after surgery to achieve P4HA1 knockdown. Western blotting confirmed the effective and specific knockdown of P4HA1 in gastrocnemius muscle tissue (Fig. 3E, Additional file 3: Fig. S3A). Immunofluorescence and Western blotting verified that adenovirus Sh-P4HA1 infection reduced P4HA1 expression in ECs (Additional file 3: Fig. S3B, C). P4HA1 knockdown significantly inhibited blood flow recovery on the 7th and 14th days in the HLI model compared to the control group (Fig. 3F, G). Immunofluorescence analysis of gastrocnemius muscle sections revealed fewer vessels in the P4HA1 knockdown group than in the control group (Fig. 3H). These findings affirm that P4HA1 knockdown exerts antiangiogenic effects both in vitro and in vivo.

### P4HA1 correlates with glycolytic metabolism reprogramming of HUVECs

**Fig. 4:**
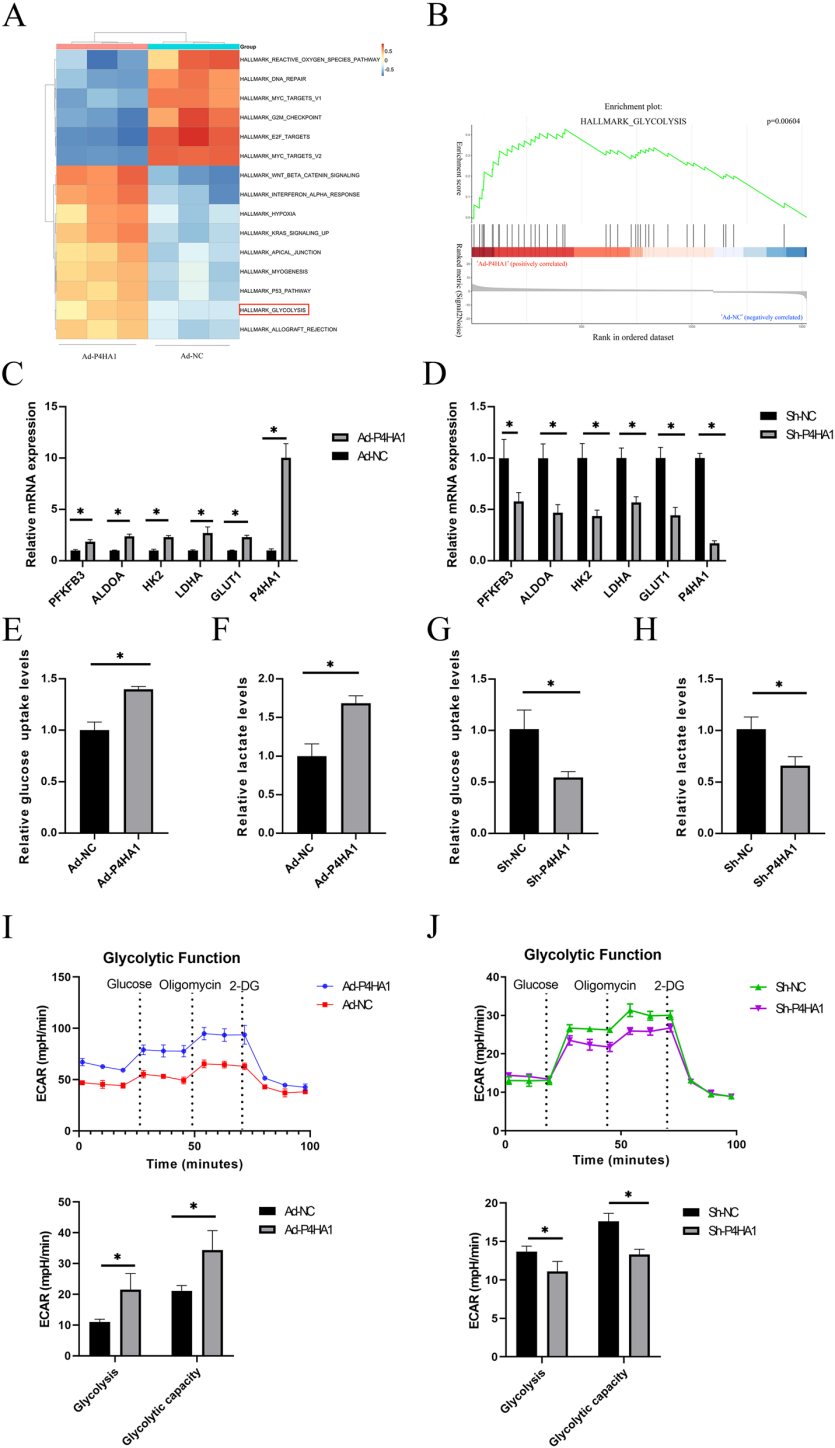
P4HA1 overexpression promotes glycolysis in HUVECs, and P4HA1 knockdown suppresses glycolysis in HUVECs. **A** GSVA based on RNA-seq data for two groups. **B** GSEA based on RNA-seq data for two groups. **C** RT-PCR analysis of the mRNA levels of PFKFB3, ALDOA, HK2, LDHA, GLUT1, and P4HA1 in HUVECs after infection with Ad-NC and Ad-P4HA1 for 24 h (*n* = 3). **D** RT-PCR analysis of the mRNA levels of PFKFB3, ALDOA, HK2, LDHA, GLUT1, and P4HA1 in HUVECs after infection with Ad-shNC and Ad-shP4HA1 for 24 h (*n* = 3). **E**, **F** HUVECs were infected with Ad-NC and Ad-P4HA1 for 24 h, **E** glucose uptake and **F** lactate production levels were determined (*n* = 3). **G**, **H** HUVECs were infected with Ad-shNC and Ad-shP4HA1 for 24 h. **G** Glucose uptake and **H** lactate production levels were determined (*n* = 3). **I** ECAR of HUVECs infected with Ad-NC and Ad-P4HA1 were measured by Seahorse XF96 Flux Analyzer. Glycolysis and glycolytic capability were statically analyzed (*n* = 3). **J** ECAR of HUVEC infected with Ad-shNC and Ad-shP4HA1 were measured by Seahorse XF96 Flux Analyzer (*n* = 3). Glycolysis and glycolytic capability were statically analyzed. Statistical differences between groups were analyzed using the Student’s t-test. *p < 0.05. *NC* negative control, *ECAR* extracellular acidification rate

Given the fundamental role of glycolysis in angiogenesis [6–8], and previous bioinformatics analyses linking P4HA1 to glycolysis in cancers [13, 22, 23], we conducted RNA-seq analysis to investigate the underlying mechanism of P4HA1-induced angiogenesis. GSVA analysis revealed that glycolysis ranked among the top 10 upregulated hallmarks in the P4HA1 overexpression group (Fig. 4A). GSEA analysis showed a similar result, indicating that P4HA1 overexpression promoted glycolysis (Fig. 4B). Thus, we concluded that P4HA1 induces angiogenesis in HUVECs by promoting endothelial glycolysis.

To delve deeper, we measured the mRNA levels of five key enzymes for glycolysis in HUVECs with P4HA1 overexpression and knockdown under normoxia. RT-PCR results demonstrated increased mRNA levels of PFKFB3, ALDOA, HK2, LDHA, and GLUT1 in the P4HA1 overexpression group and decreased levels in the P4HA1 knockdown group (Fig. 4C, D). Moreover, P4HA1 overexpression increased glucose uptake and lactate production in HUVECs under normoxia (Fig. 4E, F). P4HA1 knockdown decreased glucose uptake and lactate production in HUVECs under normoxia (Fig. 4G, H). Further validation using a Seahorse XF96 Flux Analyzer revealed that P4HA1 overexpression elevated ECAR level, glycolysis, and glycolytic capacity, whereas P4HA1 knockdown decreased ECAR level, glycolysis, and glycolytic capability under normoxia (Fig. 4I, J). Thus, we conclude that P4HA1 promotes endothelial glycolysis metabolism.

### Endothelial glycolysis inhibition reverses P4HA1-mediated angiogenesis

**Fig. 5:**
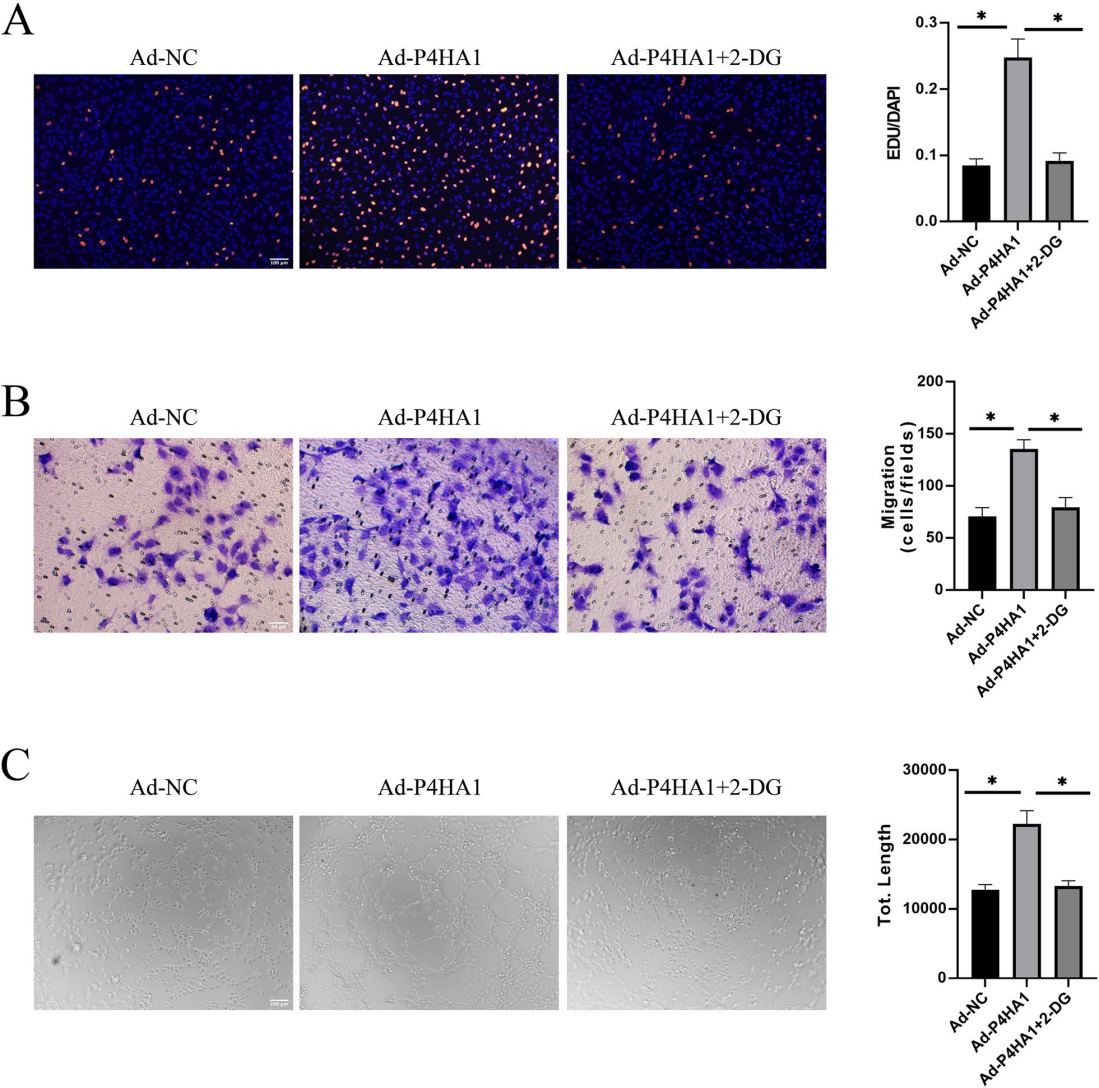
Inhibition of endothelial glycolysis counteracts P4HA1-mediated angiogenesis. HUVECs were treated with Ad-NC, Ad-P4HA1 alone, or Ad-P4HA1 and 2-DG (5 mM) for 24 h. **A** EdU incorporation assay, **B** transwell migration assay, and **C** tube formation assay were performed. Data were analyzed by one-way ANOVA followed by Bonferroni post hoc test (*n* = 3). *p < 0.05. *NC* negative control, *2-DG* 2-deoxyglucose

To confirm that P4HA1 promotes angiogenesis through glycolysis, we conducted a rescue experiment under normoxia. P4HA1-overexpressing HUVECs were treated with 5 mM 2-DG, a competitive glycolysis inhibitor, to investigate whether inhibiting endothelial glycolysis would counteract the endothelial proliferation, migration, and tube formation induced by P4HA1 overexpression. Treatment with 5 mM 2-DG resulted in a significant reduction in glucose uptake, lactate production, and cellular ATP levels in HUVECs (Additional file 4: Fig. S4A–C), indicating effective inhibition of endothelial glycolysis. Consistent with our earlier findings, P4HA1 overexpression enhanced HUVEC proliferation, migration, and tube formation (Fig. 5A–C). However, 2-DG treatment notably attenuated the P4HA1-mediated increase in the numbers of EdU-positive and migrated HUVECs (Fig. 5A, B). The tube formation assay also revealed that the increase in tube length induced by P4HA1 overexpression was abolished by glycolysis inhibition (Fig. 5C). Therefore, we conclusively affirm that P4HA1 promotes angiogenesis through the acceleration of glycolytic metabolism, and notably, inhibition of endothelial glycolysis abolishes P4HA1-induced angiogenesis.

### P4HA1 promotes glycolysis via FBP1 in HUVECs

**Fig. 6:**
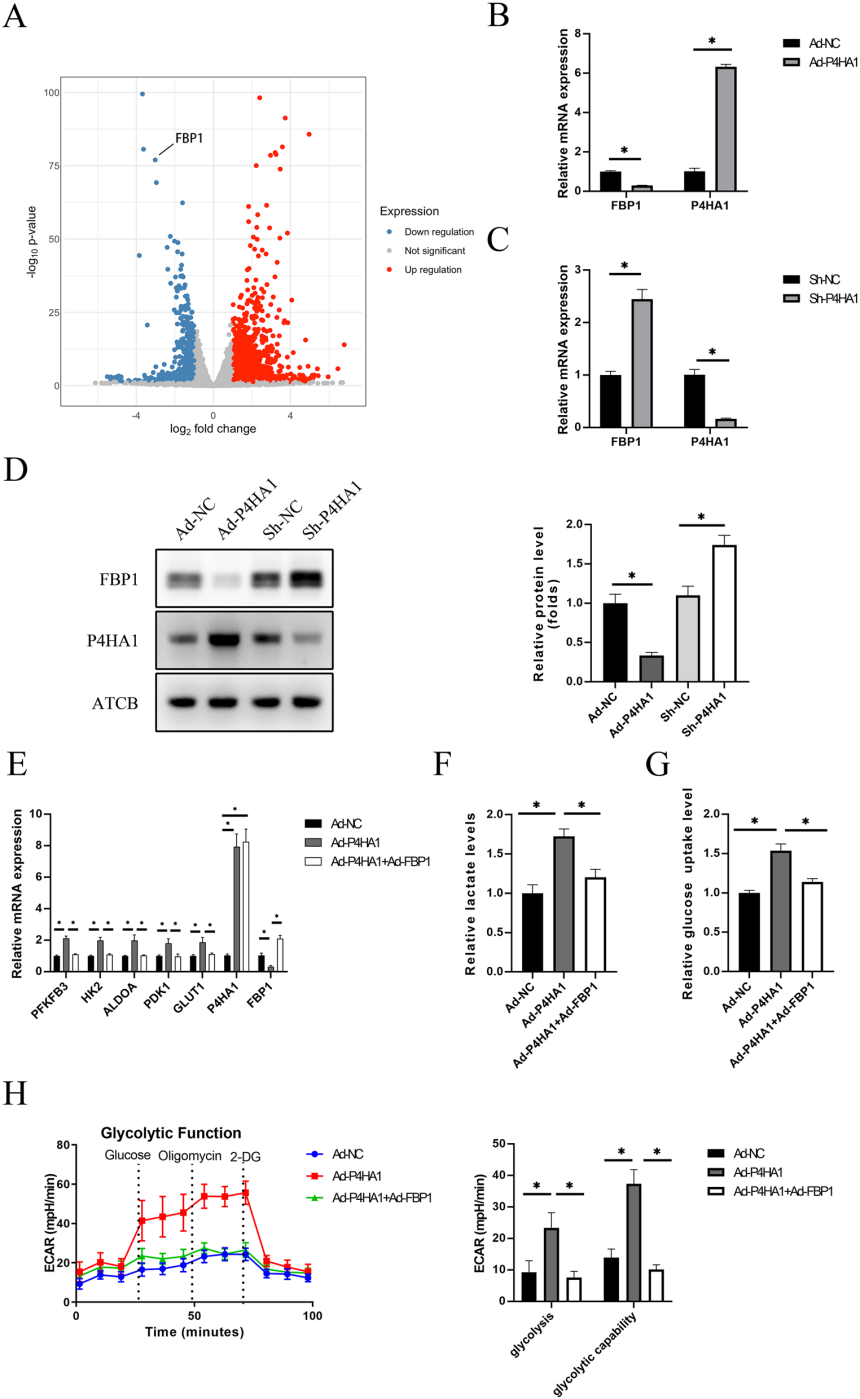
P4HA1 promotes glycolysis in HUVECs by regulating FBP1 expression. **A** Volcano plot showing differentially expressed genes in HUVECs overexpressing P4HA1 (blue, downregulated genes; red, upregulated genes; gray, genes not significantly changed). **B** RT-PCR analysis of relative mRNA levels of FBP1 and P4HA1 in HUVECs after infection with Ad-P4HA1 or Ad-NC for 24 h (*n* = 3). **C** RT-PCR analysis of relative mRNA levels of FBP1 and P4HA1 in HUVECs after infection with Ad-shNC or Ad-shP4HA1 for 24 h (*n* = 3). **D** Western blotting analysis of FBP1 and P4HA1 protein levels in HUVECs after infection with Ad-P4HA1, Ad-NC, Ad-shNC, or Ad-shP4HA1 for 24 h. Western blotting results were statically analyzed (*n* = 3). **E**–**G** After HUVECs were infected with Ad-NC, Ad-P4HA1 alone, or Ad-P4HA1 and Ad-FBP1 for 24 h, **E** mRNA levels of PFKFB3, ALDOA, HK2, LDHA, GLUT1, P4HA1, and FBP1, **F** lactate production levels, and **G** glucose uptake levels were measured and analyzed (*n* = 3). **H** ECAR of HUVECs infected with Ad-NC, Ad-P4HA1 alone, or Ad-P4HA1 and Ad-FBP1 was measured using a Seahorse XF96 Flux Analyzer. Glycolysis and glycolytic capabilities were analyzed statistically (*n* = 3). Data in **B**, **C** were analyzed using the Student’s t-test. Data in **D**–**H** were analyzed by one-way ANOVA followed by Bonferroni post hoc test. *p < 0.05. *NC* negative control

To explore how P4HA1 regulates glycolysis, we performed RNA-seq on HUVECs infected with Ad-P4HA1 or Ad-NCs under normoxia. RNA sequencing data revealed a significant decrease in FBP1 mRNA levels in the P4HA1-overexpression group (Fig. 6A). FBP1 is known to negatively regulate glycolysis in various cancers [15, 16, 24], and it can suppress glycolysis and cell proliferation by interacting with the hypoxia-inducible factor inhibitory domain of renal carcinoma [25]. RT-PCR and western blotting showed that P4HA1 overexpression decreased FBP1 mRNA and protein levels, whereas P4HA1 knockdown increased FBP1 levels under normoxia (Fig. 6B–D). Next, we investigated whether FBP1 overexpression could reverse the metabolic changes induced by P4HA1 overexpression in HUVECs under normoxia. RT-PCR results indicated that P4HA1 overexpression increased the mRNA levels of PFKFB3, ALDOA, HK2, LDHA, and GLUT1, and FBP1 overexpression partially reversed this effect (Fig. 6E). Glucose uptake and lactate assays yielded similar results, as the increased lactate production and glucose uptake observed in the P4HA1 overexpression group were attenuated by FBP1 overexpression (Fig. 6F, G). Furthermore, results from a Seahorse XF96 Flux Analyzer showed that P4HA1 overexpression promoted glycolysis and glycolytic capability in HUVECs, whereas FBP1 overexpression reversed this effect (Fig. 6H). These findings demonstrate that P4HA1 promotes glycolysis through the downregulation of endothelial FBP1.

### TET2 mediates the effect of P4AH1 on FBP1

**Fig. 7:**
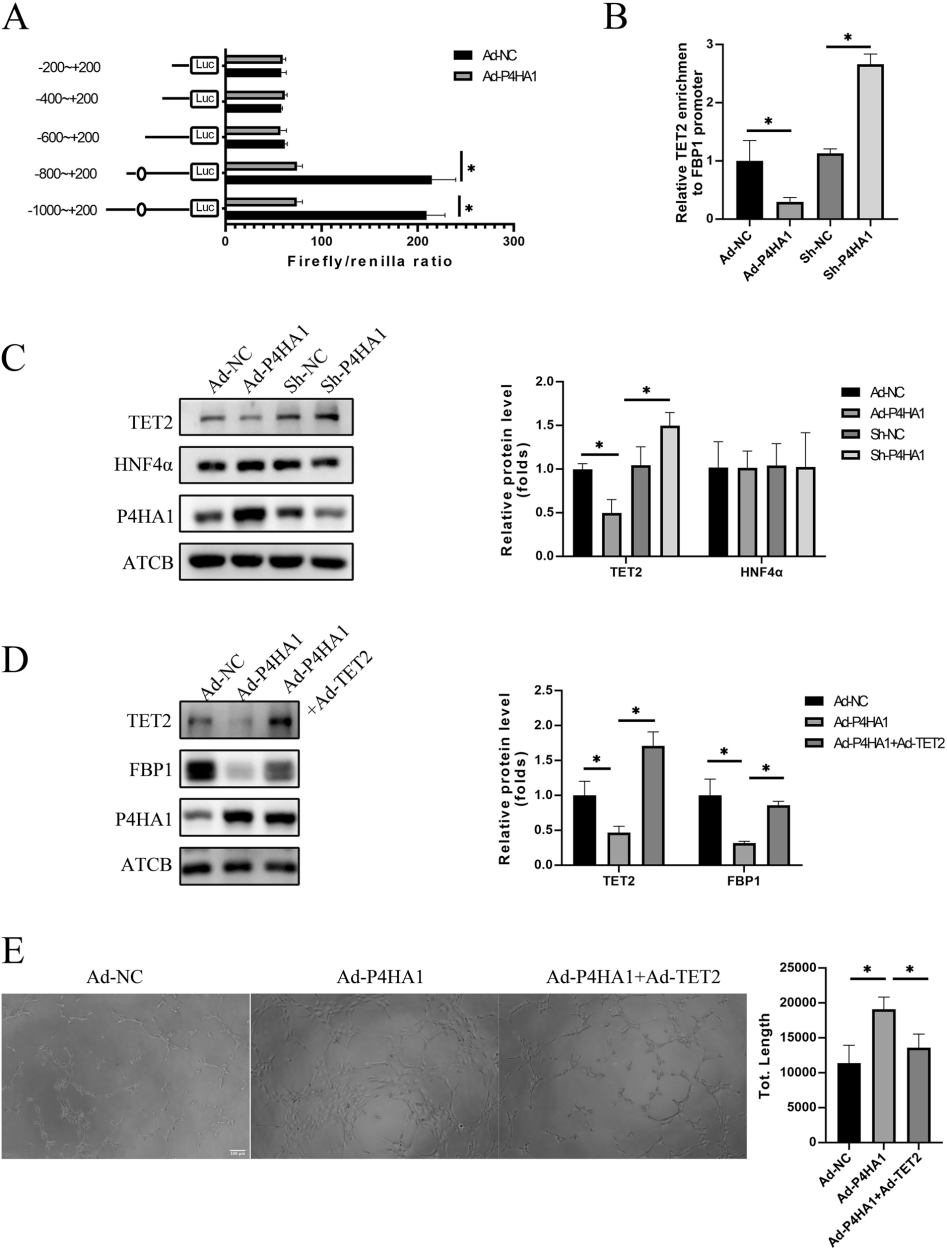
TET2 mediates P4HA1-induced FBP1 downregulation. **A** Schematic diagram of the promoter region of FBP1 in the PGL3 plasmid. Circles in the promoter represent the binding sites of HNF4α. HUVECs were infected with Ad-NC and Ad-P4HA1 and then transfected with indicated plasmids. Firefly and Renilla luciferase activities were detected 48 h later. **B** Relative TET2 recruitment to the FBP1 promoter under P4HA1 overexpression and knockdown was determined using ChIP assays. **C** Western blotting analysis of protein level of HNF4α, TET2, and P4HA1 in HUVECs after infection with Ad-NC, Ad-P4HA1, Ad-shNC, and Ad-shP4HA1 for 24 h. Western blotting results were statically analyzed (*n* = 3). **D** Western blotting analysis of protein levels of TET2, P4HA1, and FBP1 in HUVECs after infection with Ad-NC, Ad-P4HA1 alone, or Ad-shP4HA1 and Ad-TET2 for 24 h. Western blotting results were statically analyzed (*n* = 3). **E** After HUVECs were infected with Ad-NC, Ad-P4HA1 alone, or Ad-P4HA1 and Ad-TET2 for 24 h, a tube formation assay was conducted. Magnification: ×100, scale bar: 100 μm. Tube formation assay results were statically analyzed (*n* = 3). Data in **A** were analyzed using the Student’s t-test. Data in **B**–**E** were analyzed by one-way ANOVA followed by Bonferroni post hoc test. *p < 0.05. *NC* negative control

In exploring the mechanism by which P4HA1 downregulates FBP1, we designed pGL3 plasmids containing different regions of the FBP1 promoter, namely pGL3-FBP1-200 (− 200 to + 200 bp), pGL3-FBP1-400 (− 400 to + 200 bp), pGL3-FBP1-600 (− 600 to + 200 bp), pGL3-FBP1-800 (− 800 to + 200 bp), and pGL3-FBP1-1000 (− 1000 to + 200 bp). P4HA1 overexpression reduced the luciferase activities of pGL3-FBP1-800 and pGL3-FBP1-1000 but had no impact on the luciferase activities of pGL3-FBP1-200, pGL3-FBP1-400, and pGL3-FBP1-600 (Fig. 7A). Thus, we concluded that the − 800 to − 600 region of the FBP1 promoter was crucial for P4HA1-mediated FBP1 downregulation. Using the JASPAR database, we identified and validated a hepatocyte nuclear factor 4α (HNF4α)-binding site in this region. TET2 is recruited by the transcription factor HNF4α to enhance FBP1 expression [16].

We assessed the protein levels of TET2 and HNF4α under normoxia and found that P4HA1 overexpression decreased the cellular level of TET2, whereas P4HA1 knockdown increased it (Fig. 7C). Notably, neither the overexpression nor knockdown of P4HA1 affected HNF4α levels (Fig. 7C). The ChIP assay demonstrated that P4HA1 overexpression reduced the binding of TET2 to the FBP1 promoter, while P4HA1 knockdown increased it (Fig. 7B). We further investigate whether TET2 overexpression could reverse P4HA1-mediated FBP1 reduction and angiogenesis under normoxia. Our results showed that TET2 overexpression partially restored P4HA1-mediated FBP1 expression (Fig. 7D). EdU incorporation and transwell migration assays demonstrated that P4HA1 overexpression significantly enhanced the proliferation and migration capacities of HUVECs, and TET2 overexpression partially counteracted this effect (Additional file 5: Fig. S5A, B). The tube formation assay illustrated that TET2 overexpression partially reversed P4HA1-mediated angiogenesis (Fig. 7E). Therefore, we conclude that P4HA1-mediated FBP1 downregulation is dependent on TET2. P4HA1 overexpression downregulates endothelial TET2 and inhibits FBP1 transcription.

### P4HA1 regulates TET2 via α-KG

**Fig. 8:**
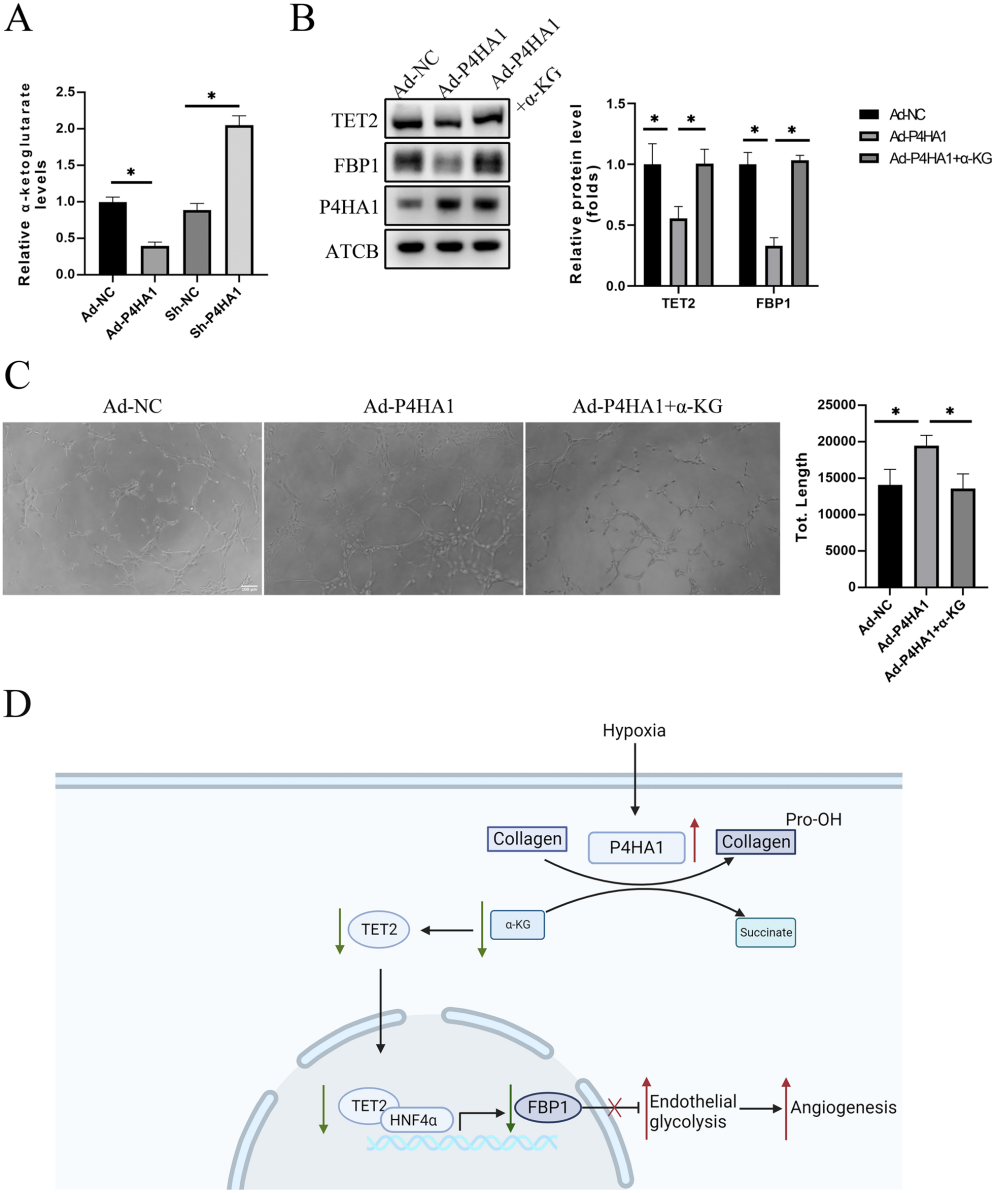
P4HA1 regulates the TET2 -FBP1 pathway by modulating cellular α-KG level. **A** Cytoplasmic α-KG was measured in HUVECs infected with Ad-NC, Ad-P4HA1, Ad-shNC, or Ad-shP4HA1 (*n* = 3). **B** Western blotting analysis of protein levels of TET2, FBP1, and P4HA1 in HUVECs after treated with Ad-NC, Ad-P4HA1 alone, or Ad-P4HA1 and 1 mM octyl-α-KG for 24 h. Western blotting results were statistically analyzed (*n* = 3). **C** After HUVECs were treated with Ad-NC, Ad-P4HA1 alone, or Ad-P4HA1 and 1 mM octyl-α-KG for 24 h, a tube formation assay was conducted. Magnification: ×100, scale bar: 100 μm. Tube formation assay results were statistically analyzed (*n* = 3). **D** Schematic model showing that P4HA1 mediates glycolysis-induced angiogenesis by suppressing the TET2-FBP1 pathway via modulation of cellular α-KG levels. Data were analyzed by one-way ANOVA followed by Bonferroni post hoc test. *p < 0.05. *α-KG* α-ketoglutarate, *NC* negative control

TET2, an α-KG-dependent dioxygenase, is influenced by cellular α-KG levels [18, 19]. P4H, an α2β2 tetrameric α-KG-dependent dioxygenase, modulates cellular α-KG levels [22, 26]. P4HA1, as a subunit of P4H, is responsible for its catalytic activity. Therefore, we inferred that P4HA1 regulates TET2 by modulating cellular α-KG levels. Under normoxia, cellular α-KG level decreased in the P4HA1-overexpression group and increased in the P4HA1-knockdown group (Fig. 8A). Treatment with 1 mM octyl-α-KG, a cell-permeable form of α-KG, partially reversed P4HA1-mediated reductions in TET2 and FBP1 under normoxia (Fig. 8B). Octyl-α-KG also alleviated P4HA1-induced HUVEC proliferation, migration, and tube formation under normoxia (Fig. 8C, Additional file 6: Fig. S6A, B). In conclusion, P4HA1 overexpression reduces the TET2 protein level by downregulating cellular α-KG.

## Discussion

This study revealed an increased expression of P4HA1 in tissues and ECs under hypoxia in the HLI model. Overexpression of P4HA1 enhanced HUVEC proliferation, migration, and tube formation, while its depletion led to endothelial dysfunction. Additionally, P4HA1 overexpression in the HLI model improved blood flow recovery, with the opposite effect observed upon P4HA1 knockdown. Mechanistically, P4HA1 increased glycolytic capacity by suppressing the TET2-FBP1 pathway in ECs, thereby promoting angiogenesis both in vitro and in vivo. P4HA1 overexpression resulted in decreased cellular α-KG levels, subsequently reducing TET2 protein levels. TET2 downregulation led to decreased FBP1 transcription, ultimately enhancing glycolysis in HUVECs. In summary, P4HA1 promoted angiogenesis through endothelial metabolism by suppressing the TET2-FBP1 pathway and modulating cellular α-KG levels (Fig. 8D).

While P4HA1 is widely expressed and highly conserved in various cell types, playing a role in glioblastoma stem-like cell transition and inducing epithelial–mesenchymal transition in glioblastoma [11, 27], our study is the first to link P4HA1 to ischemia-induced angiogenesis. Glycolysis is crucial for ATP production, and disruptions in this pathway lead to EC dysfunction [4]. Specifically, PFKFB3-mediated glycolysis promotes endothelial cell migration, proliferation, and sprouting [28]. Veys et al. highlighted the critical role of the glucose transporter GLUT1 in angiogenesis in the central nervous system during development [29]. Interestingly, bioinformatic analysis identified P4HA1 as a glycolysis-related gene [30]. Our study demonstrated that P4HA1 overexpression promoted angiogenic growth through glycolysis. Notably, Cao et al. reported that P4HA1 promoted glycolysis in pancreatic cancer cells through the P4HA1/HIF1α positive feedback loop [31]. However, our research unveiled a novel pathway by which P4HA1 promoted cellular glycolysis, namely, the P4HA1/α-KG/TET2/FBP1 pathway. P4HA1 stimulated glycolysis in tumor and normal cells through distinct pathways may be ascribed to the divergent reliance on glycolytic metabolism between these two cell types. The positive feedback loop in tumor cells enables a more rapid enhancement of glycolytic metabolism.

P4HA1-mediated collagen hydroxylation involves the consumption of α-KG and O_2_, resulting in the production of CO_2_ and succinate as byproducts [10]. P4HA1 is associated with cellular α-KG and succinate levels in breast tumor cells [22]. Our study demonstrated that overexpressing P4HA1 in HUVECs led to a reduction in cellular α-KG levels. α-KG, an intermediate product in the tricarboxylic acid cycle, plays a crucial role in oxidative phosphorylation and various physiological processes. It serves as a crucial substrate for several α-KG-dependent enzymes, including prolyl hydroxylase domain protein (PHD), P4H, and the TET family [32, 33]. Interestingly, α-KG is associated with antiangiogenic effects and delayed wound healing in diabetes [18]. Tennant et al. reported its antiangiogenic properties in solid tumors, enhancing PHD3-mediated HIF-1α hydroxylation and degradation [34]. Furthermore, α-KG supplementation increases TET2 levels, a Fe (II) and α-KG-dependent DNA dioxygenase, responsible for DNA demethylation, epigenetic reprogramming, and protein-protein interactions [19, 35]. Our study found that P4HA1-mediated α-KG depletion reduced TET2 levels, which were restored by α-KG supplementation. TET2’s effects on glycolysis have been extensively studied, revealing its role as a negative regulator. For instance, Zhang et al. reported that TET2 suppresses glycolysis in nasopharyngeal carcinoma by inhibiting pyruvate kinase M translocation to the nuclei [36]. Studies have also shown that TET2 suppresses glycolytic capability in renal carcinoma by downregulating FBP1 expression [16]. However, the relationship between TET2 expression and angiogenesis remains controversial. Tan et al. found a positive correlation between TET2 protein levels in diabetic wounds and delayed wound healing and angiogenesis [18]. Nguyen et al. demonstrated that TET2 deficiency increases angiogenesis in a lung cancer model [37]. Zhao et al. showed that TET2 depletion promotes the tube formation ability of ECs under hyperglycemia by mediating the hypomethylation of EC-specific factor roundabout 4 [38]. In contrast, Zhao et al. demonstrated that TET2 depletion inhibits the STAT3 signaling pathway and impairs angiogenesis [39]. Our study demonstrated that P4HA1-induced TET2 downregulation promoted angiogenesis by enhancing glycolysis, whereas TET2 overexpression reversed this effect.

FBP1, a rate-limiting enzyme in gluconeogenesis, exerts a negative regulatory influence on glycolysis and angiogenesis [40]. Li et al. reported that FBP1 inhibits glycolysis in renal carcinoma cells by directly binding to the HIF inhibitory domain, thereby suppressing HIF transcriptional activity [25]. Zhang et al. demonstrated that FBP1 suppresses glycolysis in nasopharyngeal carcinoma cells by inhibiting the mTOR pathway [41]. Yang et al. reported that FBP1-mediated gluconeogenesis suppresses pathological angiogenesis [42]. Our study revealed that P4HA1-induced FBP1 downregulation mediated HUVEC angiogenesis by promoting glycolysis, while FBP1 overexpression counteracted this effect. Various transcriptional and posttranslational pathways regulate FBP1 levels. Chen et al. revealed that the E3 ubiquitin ligase UBR5 promotes FBP1 ubiquitination and degradation, thereby enhancing aerobic glycolysis in pancreatic cancer [43]. Tripartite motif 47 enhances aerobic glycolysis in pancreatic cancer by binding to and promoting the ubiquitination of FBP1 [15]. Zhang et al. reported that TET2 can be recruited by the transcription factor HNF4α to activate FBP1 expression [16]. Our study demonstrated that TET2 downregulation mediated P4HA1-induced FBP1 depletion.

However, our study has some limitations. We did not elucidate the mechanism by which hypoxia induces P4HA1 upregulation. Additionally, the intramuscular adenovirus injection used for in vivo gene regulation lacked specificity for ECs. As mentioned earlier, adenovirus-mediated overexpression of P4HA1 occurred in ECs and PCs. Simultaneous immunofluorescence staining for ECs, PCs, and Flag revealed that adenovirus-mediated overexpression of P4HA1 mainly occurred in ECs and PCs of gastrocnemius muscle tissue (Additional file 7: Fig. S7A). Since PCs can influence EC function and play a crucial role in angiogenesis, it is important to rule out the possibility that P4HA1 overexpression in PCs induces angiogenesis [20]. Therefore, we established an in vivo co-grafting system of ECs and PCs through the spheroid-based angiogenesis assay to assess the influence of P4HA1 overexpression in pericytes on endothelial angiogenesis. No significant differences in vessel density of plugs were observed between the group where HUVECs were co-grafted with Ad-NC-pre-transfected PCs and the group where HUVECs were co-grafted with Ad-P4HA1-pre-transfected PCs (Additional file 7: Fig. S7B). This observation suggests that P4HA1 overexpression in pericytes did not influence angiogenesis, and the promotion of angiogenesis by P4HA1 overexpression was a localized effect in ECs.

To further clarify that in vivo angiogenesis in our experiments was mediated by the P4HA1/TET2/FBP1 pathway in ECs, we isolated ECs and non-ECs fractions from gastrocnemius muscle tissue. In the ECs fraction, P4HA1 overexpression resulted in a significant downregulation of TET2 and FBP1, while P4HA1 knockdown led to a clear upregulation of TET2 and FBP1 (Additional file 8: Fig. S8A). In the non-ECs fraction, Ad-P4HA1 infection slightly increased the P4HA1 protein level but did not change the protein levels of FBP1 and TET2 (Additional file 8: Fig. S8A). Furthermore, Ad-shP4HA1 infection did not affect protein levels of P4HA1, TET2, and FBP1 in the non-ECs fraction (Additional file 8: Fig. S8A). To further confirm that in vivo angiogenesis induced by P4HA1 overexpression was mediated by the downregulation of the endothelial TET2-FBP1 pathway, we conducted the spheroid-based angiogenesis assay. The P4HA1 overexpression group exhibited a higher vessel density in immunofluorescence-stained plug sections compared with the control group, indicating enhanced in vivo angiogenic capability (Additional file 8: Fig. S8B). Further FBP1 or TET2 overexpression based on P4HA1 overexpression reversed this effect (Additional file 8: Fig. S8B). These findings suggest that in vivo angiogenesis induced by P4HA1 overexpression was mediated by the downregulation of the endothelial TET2–FBP1 pathway, rather than other factors.

## Conclusions

Our study demonstrated that hypoxia-induced endothelial P4HA1 overexpression played an important role in post-ischemic angiogenesis by promoting endothelial glycolytic metabolism reprogramming. P4HA1 upregulation enhanced glycolysis by inhibiting the TET2–FBP1 pathway through decreasing the cellular α-KG level. These findings offer novel insights into the potential enhancement of post-ischemic angiogenesis therapy by promoting glycolytic metabolism in ECs, highlighting the therapeutic potential of P4HA1 in facilitating angiogenesis through glycolysis.

### Supplementary Information

**Additional file 1: Figure S1.** The protein levels of P4HA2, P4HA3, and P4HB in HUVECs under 0 h, 3 h, 6 h, and 12 h of hypoxia. Data were analyzed by one-way ANOVA followed by Bonferroni post hoc test (n = 3).

**Additional file 2: Figure S2.** P4HA1 is overexpressed in endothelial cells. Fourteen days after the injection of adenoviruses Ad-P4HA1 (Flag-tagged) and Ad-NC, the specified tissues were harvested from the C57BL/6J mice. (A) Western blotting analysis was used to assess Flag expression in the specified tissues. (B) Gastrocnemius muscle was fractionated into ECs and non-ECs using CD31-conjugated Dynabeads. Western blotting analysis was conducted to quantify the expression of P4HA1 in the ECs components. (C) Western blotting analysis was used to quantify the expression of Flag tag in the two components. (D) Immunofluorescence staining was utilized to visualize muscles (labeled with Myosin), blood vessels (labeled with CD31), pericyte cells (labeled with NG2), cell nuclei (labeled with DAPI), and Flag-tagged adenovirus overexpression in the gastrocnemius muscle. Magnification: ×630. Scale bar, left: 100 μm; right: 50 μm. ECs: endothelial cells, Non-ECs: non-endothelial cells.

**Additional file 3: Figure S3.** P4HA1 is knocked down in endothelial cells. Fourteen days after the injection of adenoviruses Ad-shP4HA1 and Ad-shNC, the specified tissues were harvested from the C57BL/6J mice. (A) Western blotting analysis was used to assess the P4HA1 protein level in the specified tissues. (B) Gastrocnemius muscle was fractionated into ECs and non-ECs using CD31-conjugated Dynabeads. Western blotting analysis was conducted to quantify the expression of P4HA1 in the ECs and non-ECs components. (C) Immunofluorescence staining was utilized to visualize blood vessels (labeled with CD31), cell nuclei (labeled with DAPI), and P4HA1 in the gastrocnemius muscle. Magnification: ×630, scale bar: 100 μm. ECs: endothelial cells, Non-ECs: non-endothelial cells.

**Additional file 4: Figure S4.** Treatment with 5 mM 2-DG effectively inhibits endothelial glycolysis. HUVECs were treated with vehicle or 2-DG (5 mM) for 24 h. (A) Glucose uptake, (B) lactate production, and (C) cellular ATP levels were measured. Data were analyzed using the Student’s t-test (n = 3). *p < 0.05. NC: negative control, 2-DG: 2-deoxyglucose.

**Additional file 5: Figure S5.** TET2 overexpression reverses P4HA1-mediated endothelial proliferation and migration. After HUVECs were infected with Ad-NC, Ad-P4HA1 alone, or Ad-P4HA1 and Ad-TET2 for 24 h, (A) EdU incorporation assay (Magnification: ×100, scale bar: 100 μm) and (B) transwell migration assay (Magnification: ×400, scale bar: 50 μm) were conducted. Data were analyzed by one-way ANOVA followed by Bonferroni post hoc test (n = 3). *p <  0.05. NC: negative control, EdU: 5-ethynyl-2′-deoxyuridine.

**Additional file 6: Figure S6.** Supplementation with α-KG reverses P4HA1-mediated endothelial proliferation and migration. After HUVECs were treated with Ad-NC, Ad-P4HA1 alone, or Ad-P4HA1 and 1 mM octyl-α-KG for 24 h, (A) EdU incorporation assay (Magnification: ×100, scale bar: 100 μm) and (B) transwell migration assay (Magnification: ×400, scale bar: 50 μm) were conducted. Data were analyzed by one-way ANOVA followed by Bonferroni post hoc test (n = 3). *p < 0.05. α-KG: α-ketoglutarate, NC: negative control, EdU: 5-ethynyl-2′-deoxyuridine.

**Additional file 7: Figure S7.** P4HA1 overexpression in PCs does not correlate with angiogenesis. (A) Immunofluorescence staining was utilized to visualize endothelial cells (labeled with CD31), pericyte cells (labeled with NG2), cell nuclei (labeled with DAPI), and Flag-tagged adenovirus overexpression in the gastrocnemius muscle infected with Ad-P4HA1 (Flag-tagged). Magnification: ×630. Scale bar, upper: 50 μm; lower: 25 μm. (B) Spheroids consisting of HUVECs and human microvascular pericytes pre-transfected with either Ad-NC or Ad-P4HA1 were generated. These spheroids were then incorporated into the Matrigel–fibrin matrix and subsequently injected into mice to evaluate the angiogenic capacity of HUVECs in vivo. Twenty-one days later, the mice were euthanized, and the Matrigel–fibrin plugs were harvested, embedded in paraffin, and sectioned for immunofluorescent staining targeting CD31. Representative CD31 immunofluorescence staining images of Matrigel–fibrin plug sections. Magnification: ×400, scale bar: 100 μm. Vessel density was analyzed by the Student’s t-test (n = 4). NC: negative control, PCs: pericyte cells, HUVECs: human umbilical vein endothelial cells.

**Additional file 8: Figure S8.** P4HA1 overexpression-induced angiogenesis is mediated through the TET2-FBP1 pathway in endothelial cells in vivo. (A) Western blotting analysis was used to assess the protein levels of P4HA1, FBP1, and TET2 in ECs and non-ECs fractions isolated from the gastrocnemius muscle tissue in indicated group. Statistical analysis of Western blotting was conducted with two-way ANOVA with Bonferroni post hoc test (n = 3). (B) Spheroids consisting of HUVECs pre-transfected with indicated adenoviruses (Ad-NC, Ad-P4HA1, Ad-P4HA1+Ad-FBP1, or Ad-P4HA1+Ad-TET2) were generated embedded in the Matrigel–fibrin matrix and subsequently injected into mice. Twenty-one days later, the mice were euthanized, and the Matrigel-fibrin plugs were harvested, embedded in paraffin, and sectioned for immunofluorescent staining targeting CD31. Representative images of CD31 immunofluorescence staining on paraffin sections of plugs. Magnification: ×400, scale bar: 100 μm. Vessel density was analyzed by one-way ANOVA followed by Bonferroni post hoc test (n = 4). *p < 0.05. ECs: endothelial cells, NC: negative control, Non-ECs: non-endothelial cells.

## Data Availability

The data are available from the corresponding author on reasonable request.

## Abbreviations

2-DG: 2-Deoxyglucose
α-KG: α-Ketoglutarate
ANOVA: Analysis of variance
ChIP: Chromatin immunoprecipitation
EC: Endothelial cell
ECAR: Extracellular acidification rate
EdU: 5-Ethynyl-2′-deoxyuridine
FBP1: Fructose-1,6-biphosphatase
GSEA: Gene Set Enrichment Analysis
GSVA: Gene Set Variation Analysis
HIF: Hypoxia-inducible factor
HIF-1α: Hypoxia-inducible factor-1α
HLI: Hind limb ischemia
HNF4α: Hepatocyte nuclear factor 4α
HUVECs: Human umbilical vein endothelial cells
P4H: Prolyl 4-hydroxylase
P4HA1: Prolyl 4-hydroxylase subunit alpha 1
PCs: Pericyte cells
PFKFB3: 6-Phosphofructo-2-kinase/fructose-2,6-bisphosphatase 3
PHD: Prolyl hydroxylase domain protein
PVDF: Polyvinylidene difluoride
RNA-seq: RNA sequencing
RT-PCR: Quantitative reverse transcription-polymerase chain reaction
TET2: Ten–eleven translocation 2
