# Supplementary figures and images for: Hypoxia-induced P4HA1 overexpression promotes post-ischemic angiogenesis by enhancing endothelial glycolysis through downregulating FBP1
Source: J Transl Med. 2024 Jan 18;22:74. doi: 10.1186/s12967-024-04872-x (PMC10797932; doi:10.1186/s12967-024-04872-x)

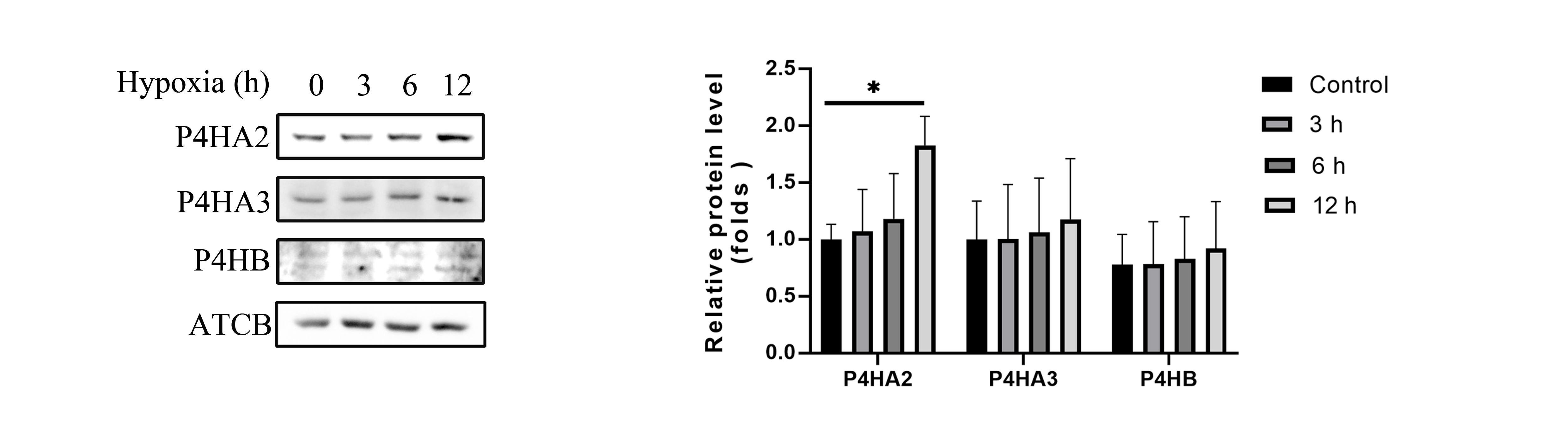

Supplement: Supplementary file 1 — Additional file 1: Figure S1. The protein levels of P4HA2, P4HA3, and P4HB in HUVECs under 0 h, 3 h, 6 h, and 12 h of hypoxia. Data were analyzed by one-way ANOVA followed by Bonferroni post hoc test (n = 3). [file 12967_2024_4872_MOESM1_ESM.jpg]

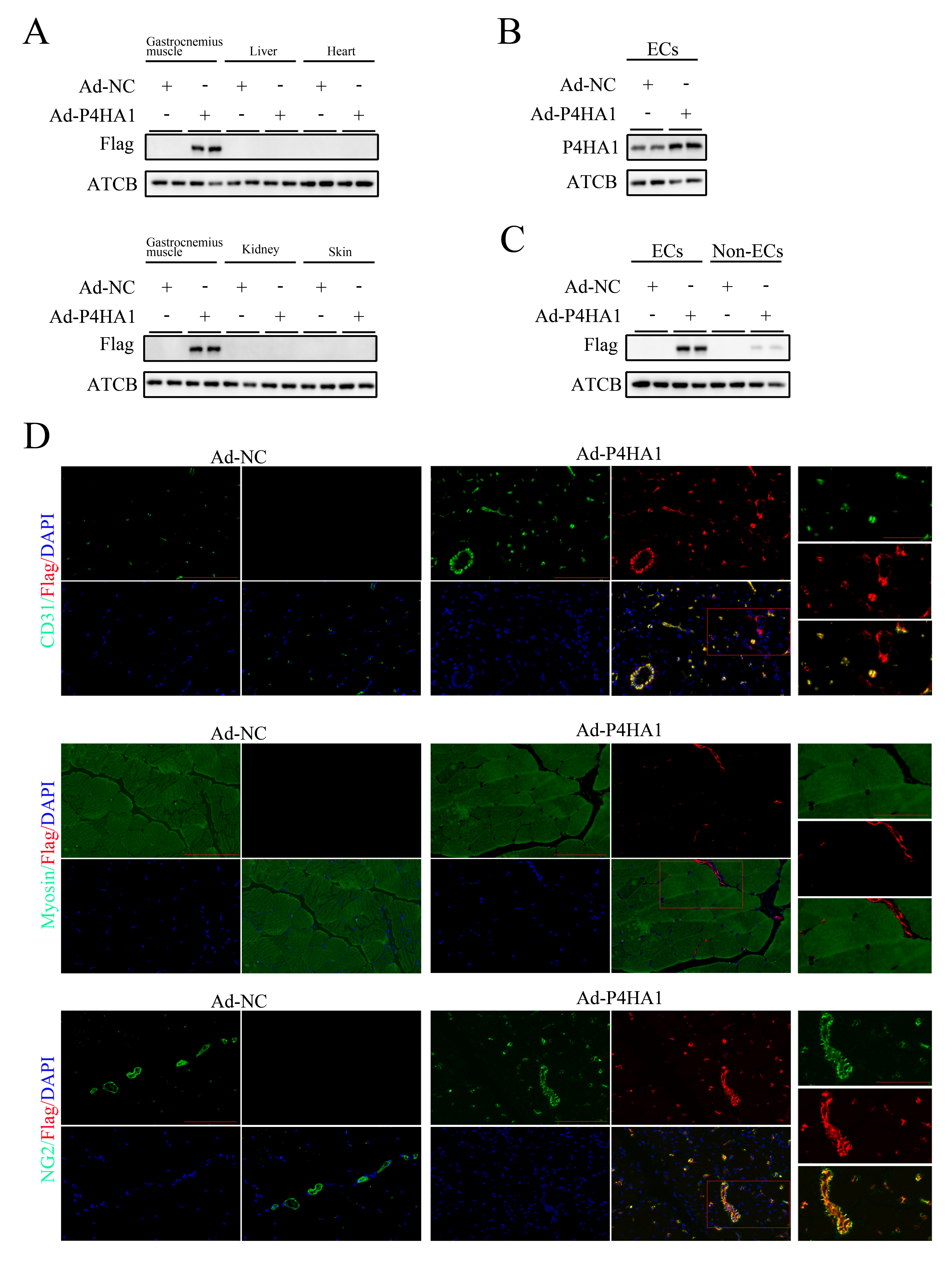

Supplement: Supplementary file 2 — Additional file 2: Figure S2. P4HA1 is overexpressed in endothelial cells. Fourteen days after the injection of adenoviruses Ad-P4HA1 (Flag-tagged) and Ad-NC, the specified tissues were harvested from the C57BL/6J mice. (A) Western blotting analysis was used to assess Flag expression in the specified tissues. (B) Gastrocnemius muscle was fractionated into ECs and non-ECs using CD31-conjugated Dynabeads. Western blotting analysis was conducted to quantify the expression of P4HA1 in the ECs components. (C) Western blotting analysis was used to quantify the expression of Flag tag in the two components. (D) Immunofluorescence staining was utilized to visualize muscles (labeled with Myosin), blood vessels (labeled with CD31), pericyte cells (labeled with NG2), cell nuclei (labeled with DAPI), and Flag-tagged adenovirus overexpression in the gastrocnemius muscle. Magnification: ×630. Scale bar, left: 100 μm; right: 50 μm. ECs: endothelial cells, Non-ECs: non-endothelial cells. [file 12967_2024_4872_MOESM2_ESM.jpg]

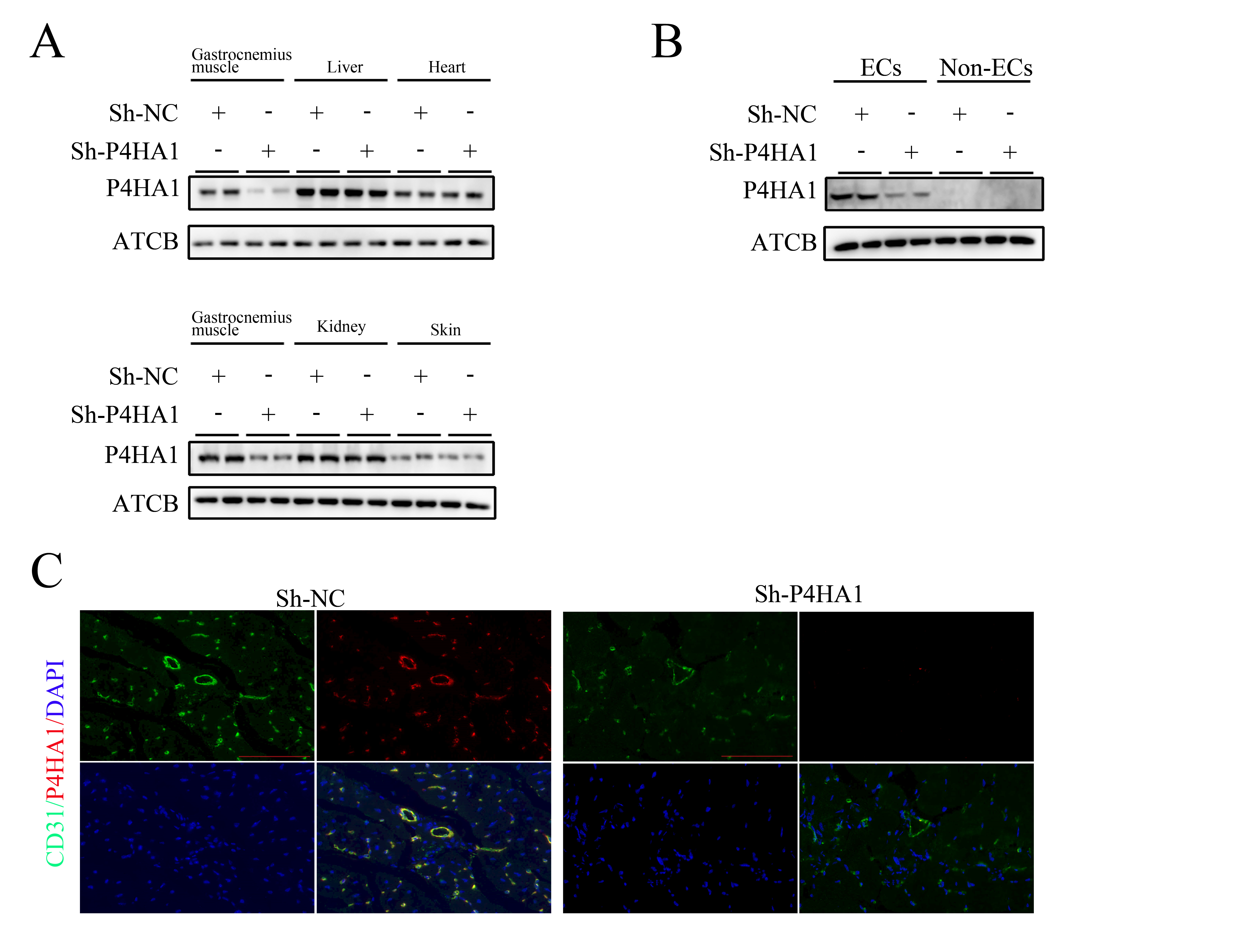

Supplement: Supplementary file 3 — Additional file 3: Figure S3. P4HA1 is knocked down in endothelial cells. Fourteen days after the injection of adenoviruses Ad-shP4HA1 and Ad-shNC, the specified tissues were harvested from the C57BL/6J mice. (A) Western blotting analysis was used to assess the P4HA1 protein level in the specified tissues. (B) Gastrocnemius muscle was fractionated into ECs and non-ECs using CD31-conjugated Dynabeads. Western blotting analysis was conducted to quantify the expression of P4HA1 in the ECs and non-ECs components. (C) Immunofluorescence staining was utilized to visualize blood vessels (labeled with CD31), cell nuclei (labeled with DAPI), and P4HA1 in the gastrocnemius muscle. Magnification: ×630, scale bar: 100 μm. ECs: endothelial cells, Non-ECs: non-endothelial cells. [file 12967_2024_4872_MOESM3_ESM.jpg]

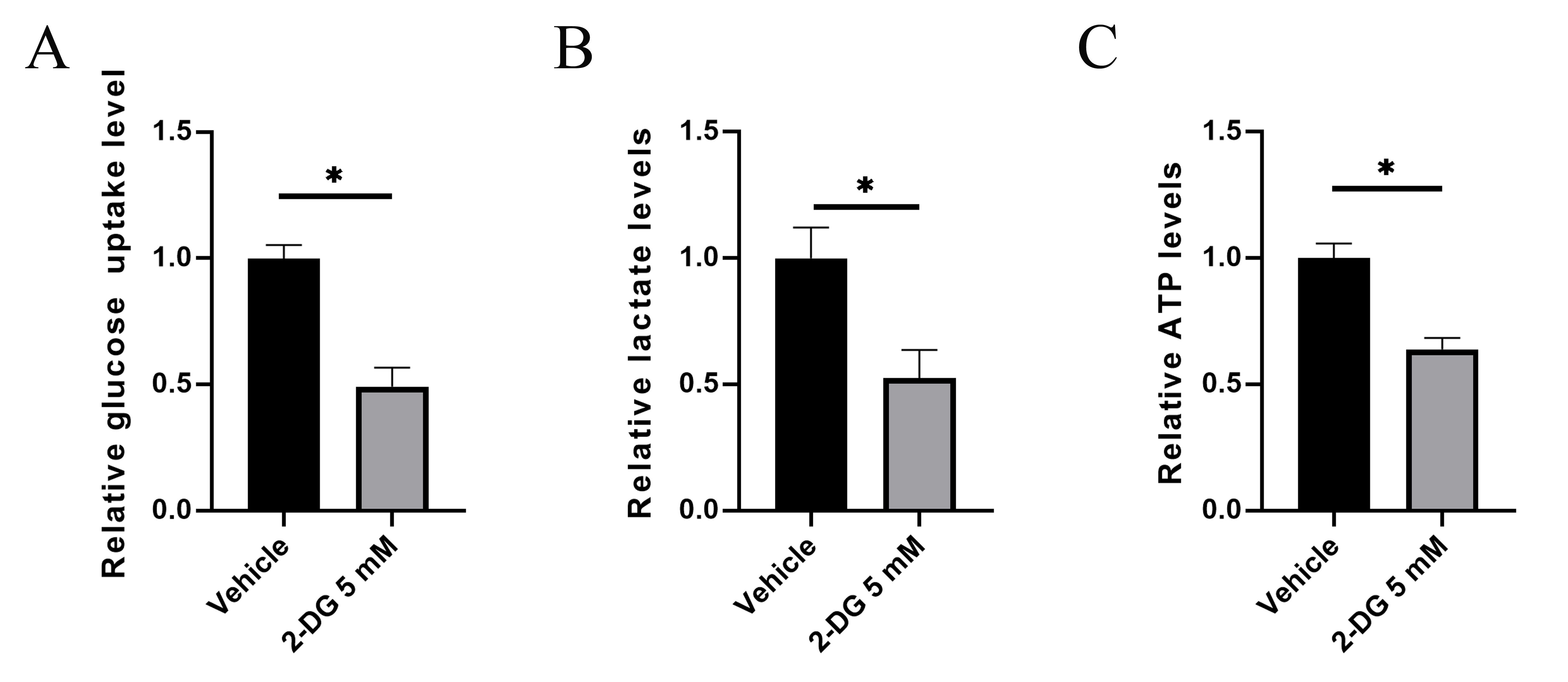

Supplement: Supplementary file 4 — Additional file 4: Figure S4. Treatment with 5 mM 2-DG effectively inhibits endothelial glycolysis. HUVECs were treated with vehicle or 2-DG (5 mM) for 24 h. (A) Glucose uptake, (B) lactate production, and (C) cellular ATP levels were measured. Data were analyzed using the Student’s t-test (n = 3). *p < 0.05. NC: negative control, 2-DG: 2-deoxyglucose. [file 12967_2024_4872_MOESM4_ESM.jpg]

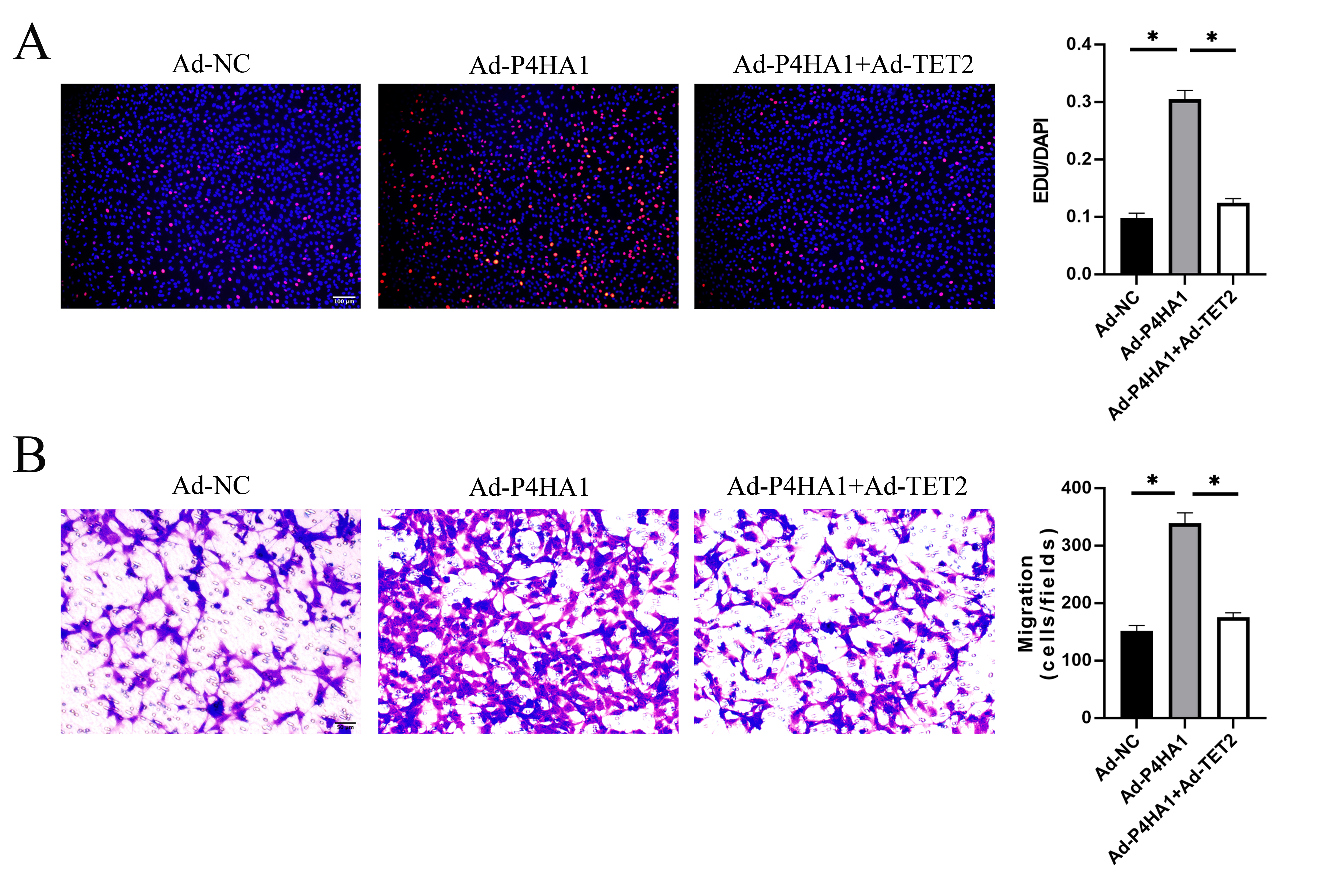

Supplement: Supplementary file 5 — Additional file 5: Figure S5. TET2 overexpression reverses P4HA1-mediated endothelial proliferation and migration. After HUVECs were infected with Ad-NC, Ad-P4HA1 alone, or Ad-P4HA1 and Ad-TET2 for 24 h, (A) EdU incorporation assay (Magnification: ×100, scale bar: 100 μm) and (B) transwell migration assay (Magnification: ×400, scale bar: 50 μm) were conducted. Data were analyzed by one-way ANOVA followed by Bonferroni post hoc test (n = 3). *p < 0.05. NC: negative control, EdU: 5-ethynyl-2′-deoxyuridine. [file 12967_2024_4872_MOESM5_ESM.jpg]

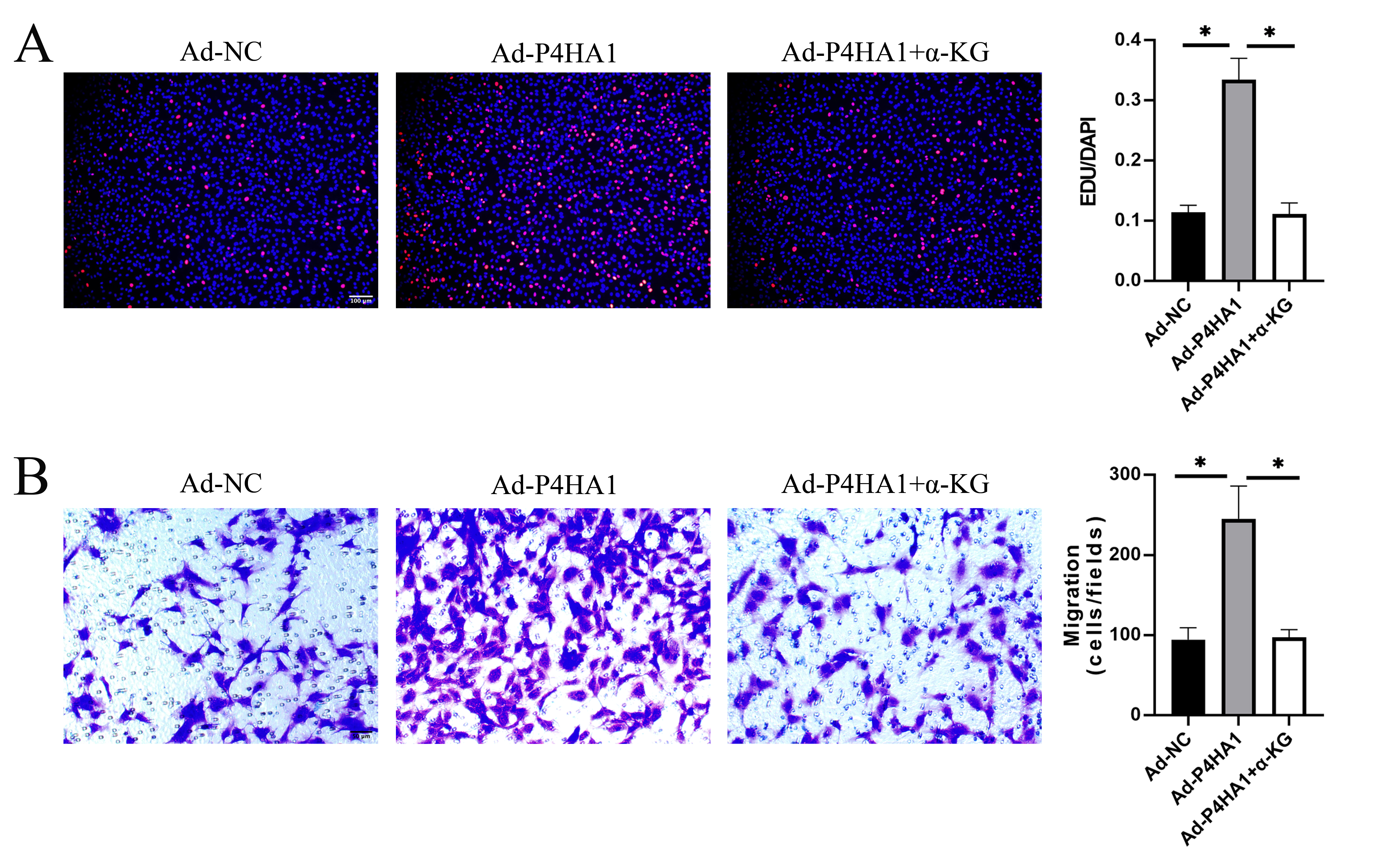

Supplement: Supplementary file 6 — Additional file 6: Figure S6. Supplementation with α-KG reverses P4HA1-mediated endothelial proliferation and migration. After HUVECs were treated with Ad-NC, Ad-P4HA1 alone, or Ad-P4HA1 and 1 mM octyl-α-KG for 24 h, (A) EdU incorporation assay (Magnification: ×100, scale bar: 100 μm) and (B) transwell migration assay (Magnification: ×400, scale bar: 50 μm) were conducted. Data were analyzed by one-way ANOVA followed by Bonferroni post hoc test (n = 3). *p < 0.05. α-KG: α-ketoglutarate, NC: negative control, EdU: 5-ethynyl-2′-deoxyuridine. [file 12967_2024_4872_MOESM6_ESM.jpg]

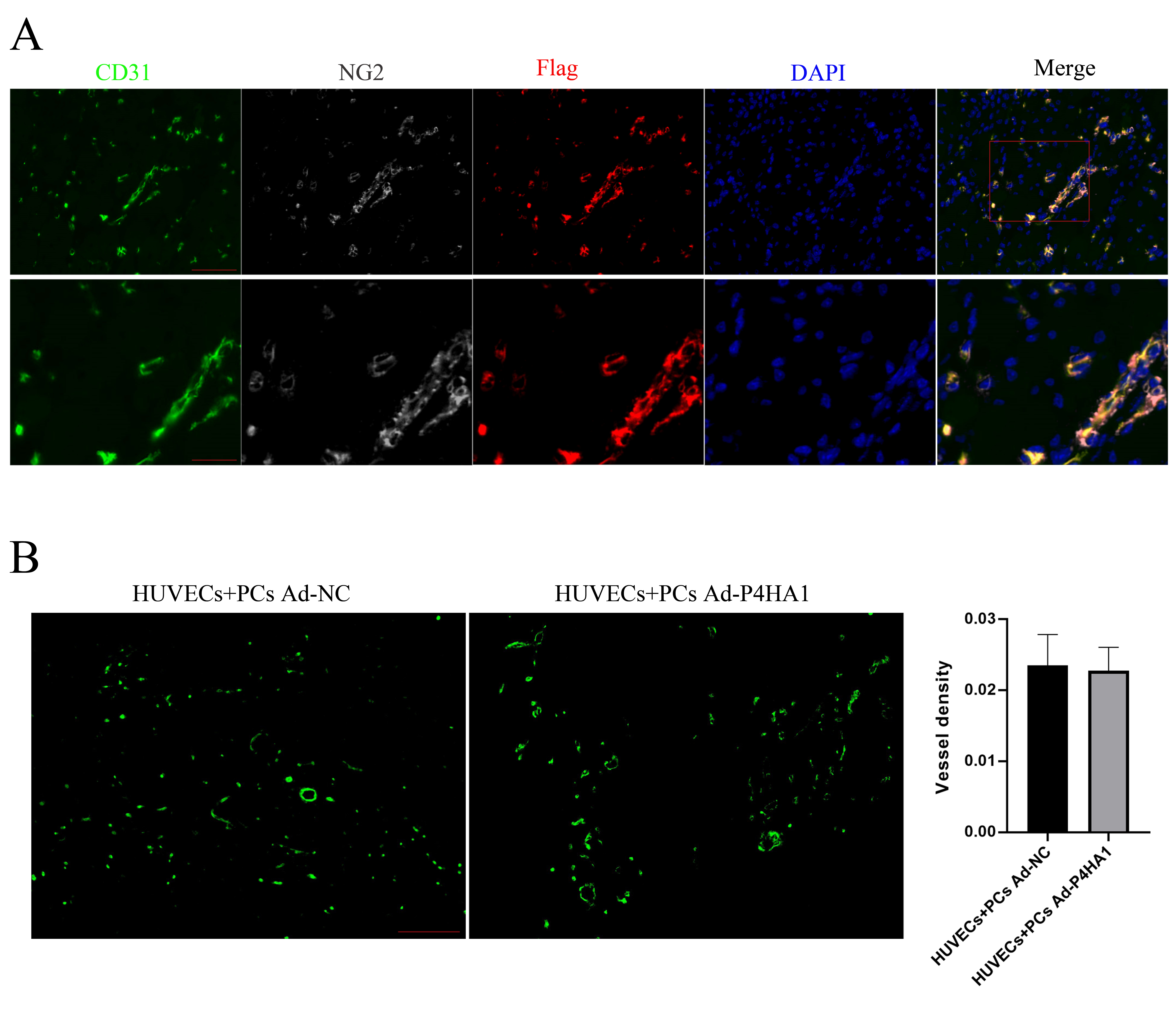

Supplement: Supplementary file 7 — Additional file 7: Figure S7. P4HA1 overexpression in PCs does not correlate with angiogenesis. (A) Immunofluorescence staining was utilized to visualize endothelial cells (labeled with CD31), pericyte cells (labeled with NG2), cell nuclei (labeled with DAPI), and Flag-tagged adenovirus overexpression in the gastrocnemius muscle infected with Ad-P4HA1 (Flag-tagged). Magnification: ×630. Scale bar, upper: 50 μm; lower: 25 μm. (B) Spheroids consisting of HUVECs and human microvascular pericytes pre-transfected with either Ad-NC or Ad-P4HA1 were generated. These spheroids were then incorporated into the Matrigel–fibrin matrix and subsequently injected into mice to evaluate the angiogenic capacity of HUVECs in vivo. Twenty-one days later, the mice were euthanized, and the Matrigel–fibrin plugs were harvested, embedded in paraffin, and sectioned for immunofluorescent staining targeting CD31. Representative CD31 immunofluorescence staining images of Matrigel–fibrin plug sections. Magnification: ×400, scale bar: 100 μm. Vessel density was analyzed by the Student’s t-test (n = 4). NC: negative control, PCs: pericyte cells, HUVECs: human umbilical vein endothelial cells. [file 12967_2024_4872_MOESM7_ESM.jpg]

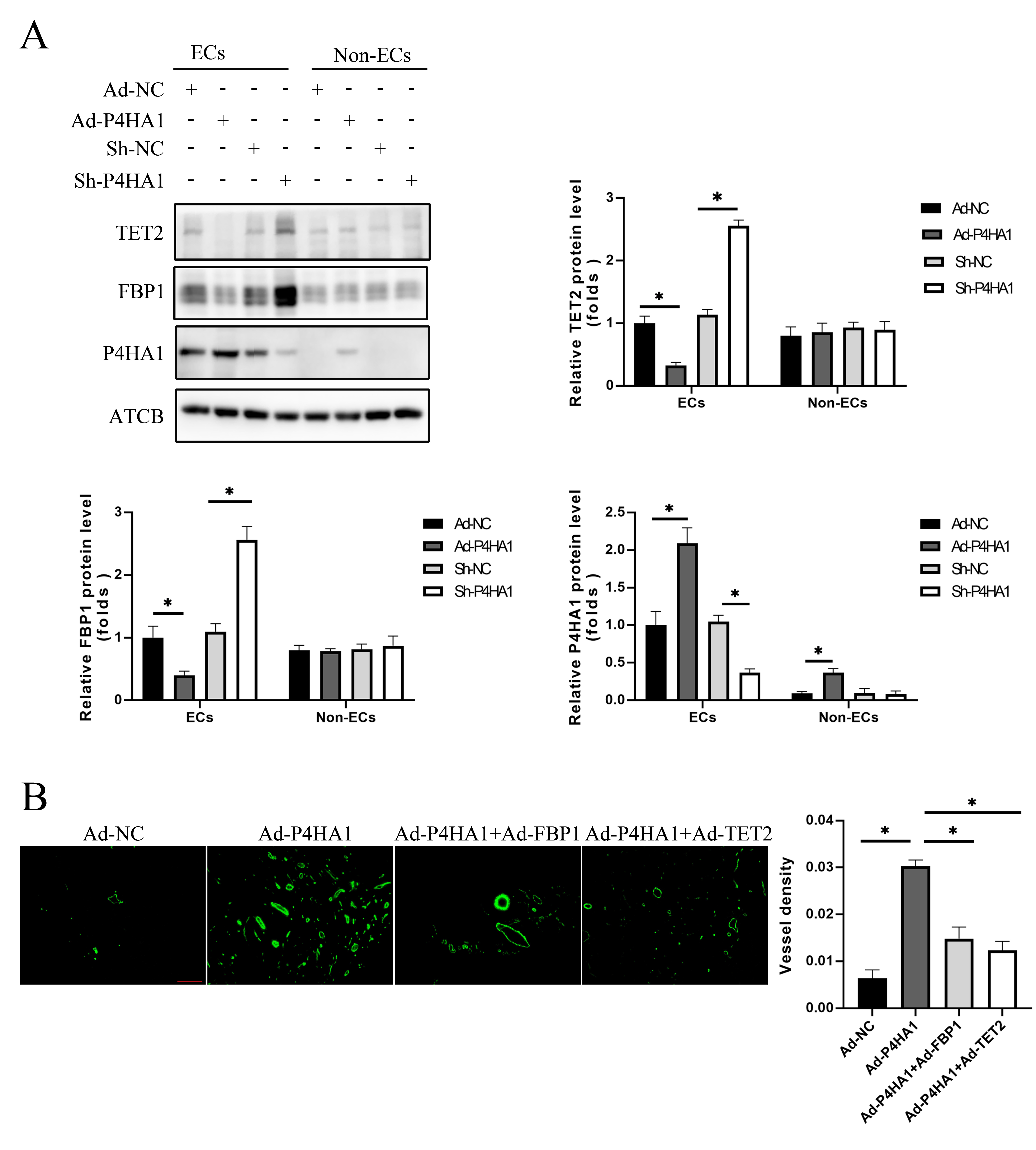

Supplement: Supplementary file 8 — Additional file 8: Figure S8. P4HA1 overexpression-induced angiogenesis is mediated through the TET2-FBP1 pathway in endothelial cells in vivo. (A) Western blotting analysis was used to assess the protein levels of P4HA1, FBP1, and TET2 in ECs and non-ECs fractions isolated from the gastrocnemius muscle tissue in indicated group. Statistical analysis of Western blotting was conducted with two-way ANOVA with Bonferroni post hoc test (n = 3). (B) Spheroids consisting of HUVECs pre-transfected with indicated adenoviruses (Ad-NC, Ad-P4HA1, Ad-P4HA1+Ad-FBP1, or Ad-P4HA1+Ad-TET2) were generated embedded in the Matrigel–fibrin matrix and subsequently injected into mice. Twenty-one days later, the mice were euthanized, and the Matrigel-fibrin plugs were harvested, embedded in paraffin, and sectioned for immunofluorescent staining targeting CD31. Representative images of CD31 immunofluorescence staining on paraffin sections of plugs. Magnification: ×400, scale bar: 100 μm. Vessel density was analyzed by one-way ANOVA followed by Bonferroni post hoc test (n = 4). *p < 0.05. ECs: endothelial cells, NC: negative control, Non-ECs: non-endothelial cells. [file 12967_2024_4872_MOESM8_ESM.jpg]
